# Decentralized nanopore genomics reveals diverse Klebsiella pneumoniae and no evidence of patient–patient transmission in a New Zealand hospital

**DOI:** 10.1099/mgen.0.001700

**Published:** 2026-04-30

**Authors:** Rhys T. White, Sarah Bakker, Megan Burton, Kristin Dyet, Juliet Elvy, Alexandra Eustace, Marissa P. Griffith, Samantha Hutton, Julianna Lees, Nathan J. Raabe, Brandon Su, Audrey Tiong, David J. Winter, Kelly L. Wyres, Max Bloomfield

**Affiliations:** 1 New Zealand Institute for Public Health and Forensic Science, Health Security, Porirua 5022, New Zealand; 2 Awanui Labs Wellington, Department of Microbiology and Molecular Pathology, Wellington 6021, New Zealand; 3 Awanui Labs Dunedin, Department of Microbiology and Molecular Pathology, Dunedin 9016, New Zealand; 4 Microbial Genomics Epidemiology Laboratory, Center for Genomic Epidemiology, University of Pittsburgh, Pittsburgh, PA 15261, USA; 5 Division of Infectious Diseases, University of Pittsburgh School of Medicine, Pittsburgh, PA 15261, USA; 6 School of Public Health, Department of Epidemiology, University of Pittsburgh, Pittsburgh, PA 15261, USA; 7 Department of Infectious Diseases, School of Translational Medicine, Monash University, Melbourne 3004, Australia; 8 Monash University, Centre to Impact AMR, Clayton 3800, Australia; 9 Department of Infection Biology, Faculty of Infectious and Tropical Diseases, London School of Hygiene, Tropical Medicine, London WC1E 7HT, UK; 10 Te Whatu Ora/Health New Zealand, Infection Services, Capital, Coast & Hutt Valley, Wellington 6021, New Zealand

**Keywords:** antibiotic resistance, infection control, phylogenetic analysis, tandem repeats, whole-genome sequencing

## Abstract

*Klebsiella pneumoniae* is a leading cause of healthcare-associated infections worldwide, yet its population structure and transmission dynamics remain largely uncharacterized in New Zealand hospitals. We conducted a 15-month prospective genomic surveillance pilot at Wellington Regional Hospital, embedding Oxford Nanopore MinION sequencing directly within the diagnostic laboratory. Clinical and screening isolates (*n*=157) underwent on-site sequencing and genotyping. Following quality control, 121 (77%) high-quality genomes (118 complete assemblies) were analysed for diversity, antimicrobial resistance (AMR), virulence loci and plasmid content and assessed for evidence of in-hospital transmission. The cohort covered many ages (0 to 95 years), with nearly half of the patients aged ≥65 years. The local *K. pneumoniae* population was highly diverse, comprising 75 distinct sequence types (STs), of which 68% were single-isolate STs. Although a few lineages recurred intermittently (e.g. ST253 and ST17), no clone showed evidence of patient–patient transmission. Plasmid reconstructions showed backbones dominated by F-type, Col, and mosaic multi-replicon elements. Acquired AMR genes were plasmid-borne in 35/118 complete genomes. Chromosomes mostly carried intrinsic determinants typical of *K. pneumoniae*: *bla*_SHV_ (intrinsic ampicillin resistance) and *fos*A and *oqx*AB (which may raise minimum inhibitory concentrations but, in their intrinsic forms, do not exceed clinical breakpoints). Few isolates carried markers of *K. pneumoniae* virulence plasmids (*iuc*/*iro* siderophores and *rmp*A/*rmp*A2; Kleborate virulence score ≥3), and none showed convergence of virulence and acquired AMR. This study shows that prospective, hospital-based nanopore sequencing is feasible in routine diagnostic settings and can deliver high-resolution genomic intelligence for infection prevention and control. In this setting, *K. pneumoniae* isolates arose from a genotypically heterogeneous background without evidence of patient–patient transmission. This pilot establishes a genomic baseline for *K. pneumoniae* in a major New Zealand hospital and supports a trigger-based framework for early detection of high-risk clones before they become established.

**Impact Statement**

Effective infection prevention and control (IPC) relies on distinguishing genuine in-hospital transmission from unrelated sporadic cases. We conducted New Zealand’s first prospective, hospital-based genomic surveillance pilot for *Klebsiella pneumoniae*, embedding portable nanopore sequencing directly into routine diagnostic workflows. This study demonstrates that decentralized genomics is practical in routine hospital workflows and can meaningfully support IPC. This ‘decentralized’ model (where DNA extraction, sequencing, first-pass analysis and reporting occur on-site) can substantially reduce turnaround times, with actionable genomic results available within <48 h of isolate recovery. This contrasts with the multi-day to multi-week delays typical of distant reference laboratory workflows, where isolates must be forwarded off-site before genomic information becomes available. While national multidrug-resistant organism surveillance appropriately prioritizes resistant phenotypes, epidemiologically important but antimicrobial-susceptible strains may be under-represented. Our on-site approach complements national systems by capturing these local events and detecting epidemiologically important strains (including antimicrobial-susceptible ones) before they become established. By showing that portable sequencing can generate timely, clinically relevant genomic insights at the point of care, this work provides a practical model for delivering timely genomic intelligence to strengthen IPC in healthcare settings.

## Data Summary

The study sequences are available in the National Center for Biotechnology Information (NCBI) under BioProject accession number PRJNA1332379. The raw sequence read data generated in this study have been deposited in the NCBI sequence read archive (SRA (https://www.ncbi.nlm.nih.gov/sra) under accession numbers SRR35539550 to SRR35539706. The complete assemblies have been deposited in GenBank under the accession numbers JBREKV000000000 to JBREPI000000000. As of July 2025, GenBank is transitioning to a unified processing system for all prokaryotic and eukaryotic genomes; these records will remain accessible and automatically incorporated into the new platform. The software used to analyse raw sequence reads for polymorphism discovery and whole-genome sequencing-based phylogenetic reconstruction and plasmid analysis is available as described in the ‘Methods’ section. The authors confirm that all supporting data protocols have been provided in the article or supplementary data files.

## Introduction

Antimicrobial resistance (AMR) accounted for at least 1.27 million deaths globally in 2019, with *Klebsiella pneumoniae* among the six leading pathogens [1]. Mortality is often high; for example, in a tertiary-care centre in India, nearly half of patients with bloodstream infections caused by carbapenem-resistant *K. pneumoniae* died [2]. Beyond the health burden, the projected economic impact of AMR is substantial. Global productivity losses are projected to reach US$100 trillion and a reduction of up to 3.8% in global gross domestic product by 2050, potentially pushing 28 million people into poverty if AMR remains unchecked [3,4]. These impacts are unlikely to be evenly distributed, with low- and middle-income countries expected to bear a disproportionate share of the health and economic consequences. *K. pneumoniae*, therefore, remains a critical threat to global health security, combining high disease burden with escalating resistance [5,7].

Since its first recognition as a cause of pneumonia in 1882 [8], *K. pneumoniae* has become a prominent opportunistic pathogen in healthcare settings [9,10]. *K. pneumoniae* is often associated with urinary tract infections, pneumonia, wound infections and sepsis [10,14]. Colonization of the gut, nasopharynx and skin is frequent [13], and in intensive care and oncology patients, intestinal carriage of *K. pneumoniae* raises the risk of hospital-associated infection fourfold [15,16]. Genomic research confirms that strains colonizing the gut are a major source of infection [15,16]. In long-term care facilities, increased gut abundance of colonizing *K. pneumoniae* has been associated with subsequent bloodstream infection [17], indicating that the intestinal reservoir can act as a source of opportunistic infection, particularly when colonization involves multidrug-resistant strains.

At the global level, surveillance remains inconsistent, and coverage varies. While international frameworks emphasize strengthening and prioritizing AMR monitoring, much of the evidence still derives from incomplete or fragmented testing [7]. In 2025, the World Health Organization Global Antimicrobial Resistance and Use Surveillance System (GLASS) report estimated that (in 2023) ~46% of reporting countries had implemented all five core surveillance components, and overall national data completeness was ~54% [18]. Notably, AMR data from New Zealand (known as Aotearoa in the Māori language) were not included in the 2025 GLASS report [18], highlighting under-representation within the Western Pacific region and aligning with an ongoing transition towards a more integrated national surveillance system [19]. This can limit the availability of timely, locally representative signals on pathogen dynamics. To help close this evidence gap, prospective, high-resolution genomic surveillance that includes both colonization (carriage) and clinical isolates can support near-real-time detection and tracking of high-risk lineages and the resistance and virulence genes they carry. Crucially, it complements (not replaces) national data integration and GLASS AMR reporting by supplying the genomic context needed to interpret trends and surface early signals.

Although *K. pneumoniae* is the most clinically well-known member of the *Klebsiella* genus, some standard diagnostic systems frequently fail to distinguish *K. pneumoniae* from closely related taxa [20,22]. Genomic analyses have, therefore, broadened the definition to the *K. pneumoniae* species complex (KpSC), which includes *K. pneumoniae*, *Klebsiella quasipneumoniae*, *Klebsiella africana*, *Klebsiella variicola* and *Klebsiella quasivariicola*; these differ by ~3–4% nucleotide divergence across core chromosomal genes [14,22,31]. The population structure of the KpSC is complex, with more than 1,400 sequence types (STs) catalogued globally and antigenic heterogeneity at the capsule (K-antigen) and lipopolysaccharide (O-antigen) loci [12,14, 30, 32,38]. Even within a single hospital, clinical collections typically comprise dozens of distinct STs [13,39]. Importantly, most opportunistic infections emerge from this diverse background, but a few specific epidemic multidrug-resistant clones, such as clonal group (CG)15, CG258 and CG307, have spread globally as dominant carriers of extended-spectrum beta-lactamases (ESBLs) and carbapenemases [12,40]. In contrast, hypervirulent clones (most prominently CG23, but also CG65 and CG86) are generally drug-susceptible but enriched for virulence loci. These include siderophore systems like aerobactin (encoded by *iuc*), salmochelin (encoded by *iro*) and yersiniabactin (encoded by *ybt*), as well as the mucoid regulators *rmp*A/*rmp*A2 [12,41, 42]. These hypervirulent clones can cause invasive community-acquired infections in otherwise healthy hosts, reported globally but most commonly within Asia [43]. Unlike multidrug-resistant clones, hypervirulent lineages tend to maintain comparatively stable genomes, less frequently acquiring AMR genes and showing far lower accessory gene diversity, highlighting distinct evolutionary trajectories [14,32, 44]. This ecological divide (i.e. widespread opportunistic diversity contrasted with a few epidemic multidrug-resistant or hypervirulent clones) complicates infection prevention strategies and underscores the value of whole-genome sequencing (WGS) for resolving sporadic infections, detecting/confirming outbreaks and identifying emerging high-risk lineages that pose clinical threats [12,14, 45,47].

Against this backdrop, WGS is recognized as a critical component of pathogen surveillance [45,48,54] and is indispensable for understanding the epidemiology of *K. pneumoniae* [12,14]. However, most hospital-based studies to date have been retrospective, reliant on centralized short-read sequencing and disproportionately focused on resistant isolates [12,13, 39, 45, 55]. While these approaches provide valuable insights, they are limited in their ability to guide real-time infection control. Portable nanopore sequencing devices enable prospective, decentralized analysis of these genomes within diagnostic laboratories [22,45, 56, 57]. With current super-accuracy basecalling and improved assembly-and-polishing workflows, Oxford Nanopore sequencing now achieves reliable performance for core applications, including multilocus sequence typing (MLST), AMR and virulence gene detection, serotyping and increasingly single-nucleotide variant (SNV) and insertion or deletion (indel) calling, supporting the feasibility of frontline implementation [22,45, 56,60].

New Zealand provides a distinctive setting for evaluating proactive genomic surveillance of *K. pneumoniae*. Geographic isolation, historically low rates of carbapenem resistance and limited penetration of high-risk global clones create an opportunity to assess whether hospital transmission drives infections in the absence of entrenched epidemic lineages. National surveillance indicates that carbapenemase-producing *Enterobacterales* remain uncommon, with most cases linked to overseas travel, and few local transmission events detected to date [61,62]. New Zealand has piloted a decentralized model of pathogen sequencing, deploying Oxford Nanopore Technologies devices directly into frontline hospital laboratories [56,63]. This model can enable rapid, prospective sequencing of target pathogens on site, with centralized bioinformatic support available when needed [22], and offers a scalable template for smaller hospitals worldwide. To our knowledge, prospective real-time sequencing has not previously been applied to characterize *K. pneumoniae* in a New Zealand hospital systematically. This setting tests the technical feasibility of decentralized sequencing to establish a local genomic baseline and flag early introductions; if applied across multiple sites, the same approach should detect incursions before they become endemic.

Here, we report, to our knowledge, the first prospective genomic surveillance of *K. pneumoniae* in New Zealand, undertaken at Wellington Regional Hospital using onsite MinION sequencing. Over 15 months, clinical isolates were sequenced to establish a high-resolution baseline of the local population structure. We aimed to assess whether patient *K. pneumoniae* cases showed genomic evidence of sustained in-hospital transmission or instead a polyclonal background with limited relatedness between cases. By integrating data on lineage diversity, resistance and virulence genotypes and temporal trends, we provide insights into the ecology of *K. pneumoniae* in a low-resistance environment. More broadly, this work provides a practical template for data-driven, locally prioritized real-time sequencing with relevance well beyond New Zealand.

## Methods

### Sample collection/study design

This was a 15-month prospective sequencing pilot of all non-duplicate *K. pneumoniae* isolates from both diagnostic (‘clinical’) and infection-prevention screening (‘screening’) specimens collected at Wellington Regional Hospital between January 2022 and April 2023. Clinical isolates were organisms recovered from diagnostic specimens submitted to investigate suspected infection in symptomatic patients. Screening isolates were organisms recovered from specimens collected to detect asymptomatic carriage under local infection-prevention protocols (e.g. rectal/perineal swabs). Screening was not universal and was restricted to defined risk-based indications on hospital admission, including (i) recent hospitalization overseas or in another New Zealand hospital within the preceding 12 months, (ii) recent travel to South or Southeast Asia or the Indian subcontinent within the preceding 12 months or (iii) the presence of a long-term indwelling urinary catheter.

In addition to these admission-based indications, limited reactive contact screening could also be undertaken under local infection prevention and control (IPC) protocols after defined exposure events (e.g. shared bathroom exposure to a patient with an ESBL-producing *Klebsiella* for >24 h); however, no routine universal admission screening programme for *K. pneumoniae* was in place. As a result, screening isolates represent a targeted subset of patients rather than the general inpatient population. Repeat isolates within three months in the same patient were excluded. Cases were classified as community- or hospital-onset based on time from hospital admission, with a 72-h threshold used to define hospital-onset cases to increase specificity for potential healthcare-associated acquisition. Isolates initially identified as *K. pneumoniae* by routine diagnostics but resolved by WGS as other *Klebsiella* species (e.g. *K. variicola* [22]) were excluded from downstream analyses and are not reported here.

**Fig. 1.**
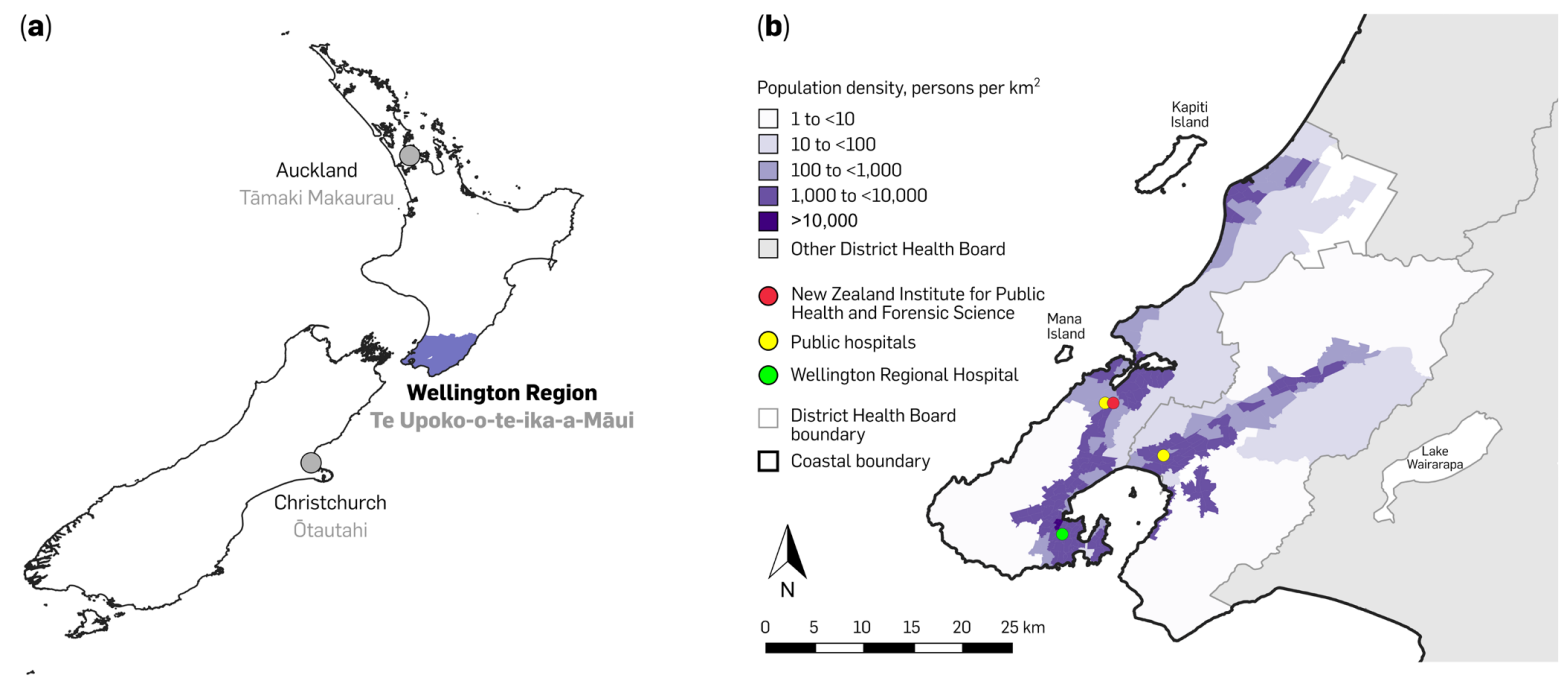
Geographic and demographic context of the Wellington Region, New Zealand. (**a**) Location of the Wellington Region (Te Upoko-o-te-Ika-a-Māui) in the lower North Island (Te Ika-a-Māui), shown relative to Auckland (Tāmaki Makaurau) and Christchurch (Ōtautahi). (**b**) Population density by Statistical Area 2 based on mid-year (30 June) population estimates (https://www.stats.govt.nz/topics/population/, accessed on 14 March 2025). District Health Board (DHB) boundaries are indicated, population shown is within the Capital and Coast and Hutt Valley DHB. Symbols mark key facilities: the New Zealand Institute for Public Health and Forensic Science (red), Wellington Regional Hospital (green) and other public hospitals (yellow).

Wellington Regional Hospital provides tertiary care for ~500,000 people across the lower North Island (known in te reo Māori as Te Ika-a-Māui), New Zealand (Fig. 1). Clinical microbiology testing is performed by Awanui Labs (Wellington, New Zealand), an ISO15189-accredited facility that processes ~300,000 samples annually. Bacterial identification is undertaken using Vitek^®^ MS PRIME (bioMérieux, database v3.3.0). Antimicrobial susceptibility testing of *Enterobacterales* is performed on the Vitek^®^ 2 (bioMérieux, Marcy-l’Etoile, France) with the AST-N311 card and interpreted according to the European Committee on Antimicrobial Susceptibility Testing guidelines [64]. Vitek^®^ 2 reports categorical susceptibility interpretations [susceptible/intermediate/resistant (S/I/R)] derived from discrete antimicrobial concentration steps, which are not equivalent to reference broth microdilution minimum inhibitory concentration (MIC). Where we refer to elevated non-susceptibility, we mean a shift from S to I/R in the Vitek^®^ 2 categorical call.

This work is a collaboration between the New Zealand Institute for Public Health and Forensic Science (PHF Science) (formerly the Institute of Environmental Science and Research) and Awanui Labs Wellington. PHF Science provided sequencing oversight, bioinformatics and genomic analyses. Within New Zealand’s public-health system, PHF Science is responsible for national notifiable disease and AMR surveillance and supports government agencies with laboratory science, AMR genomics, infectious disease surveillance and microbiology/public health expertise, delivered via secure data infrastructure and integrated outbreak-response programs.

### Extraction of DNA for nanopore sequencing

At Awanui Labs Wellington, *Klebsiella* spp. isolates were subcultured on 5% sheep blood agar and incubated for 24 to 48 h at 35 °C to ensure purity. A loopful of growth was suspended in PBS and incubated at 37 °C for ≥30 min before automated extraction on the MagNA Pure 96 platform (Roche, Switzerland; DNA and Viral NA Small-Volume Kit with Pathogen-Universal-200 Extraction protocol), following the established laboratory workflows [22,63]. DNA extracts were stored at 4 °C until sequencing. The length of storage time varied depending on when the samples were received relative to the next scheduled sequencing run.

Library preparation was carried out using 50 ng input DNA and the Oxford Nanopore Rapid Barcoding Kit 96 (SQK-RBK110-96, Oxford, UK), as per the manufacturer’s instructions. Sequencing was performed on MinION devices using R9.4.1 flow cells (FLO-MIN106), with runs typically multiplexing 10 to 18 isolates and lasting 20 to 40 h under MinKNOW v22.10.10. Library preparation and flow cell loading required ~4 h of hands-on time, with sequencing proceeding as a walk-away process, resulting in a technical turnaround time of ~24 to 48 h from DNA extraction to sequencing completion [63]. In practice, isolates were batched for routine surveillance and sequenced approximately fortnightly, resulting in an operational turnaround time of up to ~2 weeks. Urgent samples, however, could be prioritized and processed within a few days when required [22].

### Retrospective basecalling and quality control of the nanopore sequence data

At PHF Science, nanopore raw data were processed and quality checked as described in our earlier study [22]. In brief, Fast5 files were converted to Pod5 format using pod5 v0.3.2 (https://github.com/nanoporetech/pod5-file-format, accessed on 01 October 2025) and basecalled using Dorado v0.4.3 (https://github.com/nanoporetech/dorado, accessed on 01 October 2025) using the ‘dna_r9.4.1_e8_sup@v3.6’ super accuracy model, with a batch size of 2008 and a chunk size of 1,000, while other parameters were kept at their default settings. Basecalling was performed on an NVIDIA A100-PCIE-80GB graphics processing unit. An overview of the analytical workflow, including genome reconstruction, genotyping and comparative genomics steps, is provided in Fig. S1 (available in the online Supplementary Material). Basecalled read quality was assessed using the Nanopack v1.6.0 suite [65] (i.e. NanoStat v1.6.0, NanoFilt v2.8.0). Low-quality regions were trimmed (52 bp from each end to minimize end-of-read error artefacts). Very low-accuracy reads (mean quality score <Q7; equivalent to ~80% per-base accuracy) were discarded, and taxonomic profiling was conducted with Kraken v2.1.3 [66] (with default parameters) against the National Center for Biotechnology Information (NCBI) Reference Sequence (RefSeq) Standard database (https://benlangmead.github.io/aws-indexes/k2, accessed on 01 October 2025) [67]. This database contained references for archaea, bacteria, humans, viruses, plasmids and the ‘UniVec core’ subset of the UniVec database (a database of vector, adaptor, linker and primer sequences).

### *De novo* assembly of the quality-filtered nanopore sequence data

Assemblies were generated using Autocycler v0.5.2 with the read type set to ‘ont_r9’ [68]. Autocycler executed several assembly algorithms (Canu v2.3 [69], Flye v2.9.6 [70], miniasm v0.3 [71], NextDenovo v2.5.2 [72] and Raven v1.8.3 [73]) and then applied graph-based clustering and refinement to derive a high-confidence consensus assembly. The resulting consensus underwent three rounds of polishing: nanopore reads were aligned back to contigs with minimap2 v2.24 [74,75] (configured with the ‘map-ont’ preset), and SNVs and indels were corrected with Racon v1.4.3 [76]. Racon was set up with parameters: ‘--match 8’ for match score, ‘--mismatch −6’ for mismatch score and ‘--gap −8’ for gap penalty. Filtered nanopore reads were first aligned to the draft assembly using minimap2, and the resulting alignments (PAF format) were used as input for racon. This procedure was repeated for three iterative polishing cycles in total: after each round, reads were realigned to the newly polished assembly, new alignments were generated and Racon was reapplied with the same parameter set. Consistent use of parameters across all rounds ensured uniform error correction throughout the polishing process. Final chromosomal and plasmid assemblies were reoriented with dnaapler v1.3.0, using the ‘all’ function to set the chromosome start at *dna*A and plasmid sequences at *rep*A [77].

### Genotypic characterization of assembled genomes

Reoriented assemblies (FASTA format) were submitted to the NCBI GenBank repository, where they were automatically annotated using the Prokaryotic Genome Annotation Pipeline (PGAP) [78,80]. All annotations reported in this study, therefore, reflect the standardized NCBI PGAP process applied at the time of submission. FASTA assemblies were also uploaded to the PathogenWatch platform (https://pathogen.watch, accessed on 01 October 2025) for standardized *Klebsiella* spp. analyses. Species identification was performed using Speciator v4.0.0 (https://github.com/pathogenwatch-oss/speciator, accessed on 01 October 2025) [81]. Downstream analyses were restricted to *K. pneumoniae*; genomes resolved as other members of the KpSC (e.g. *K. variicola*) were excluded as described above. MLST lineages were assigned according to the *Klebsiella* PasteurMLST sequence definition database (version 2025-09-01-kpneumoniae) [37] hosted on BIGSdb v1.47.0 [82]. Capsule (K) and O-antigen loci were predicted with Kaptive v3.1.0 [83,85], including locus type, capsule or O-serotype prediction and *wzi* allele assignment. Acquired AMR genes were identified using Kleborate v3.2.4 [12]. Genomes were also screened with AMRfinderplus v3.12.8 with database v2024-01-31.1 to cross-validate AMR calls and capture additional determinants [86]. Plasmid replicon typing was performed by screening assemblies against the PlasmidFinder database [87,88]. For comparison, plasmid groups were assigned based on MOB-suite v3.1.7 [89,90] and Mash nearest-neighbour relationships [91]. Virulence-associated loci (yersiniabactin, colibactin, aerobactin, salmochelin and *rmp*A/*rmp*A2) were also detected with Kleborate, with virulence scores calculated according to standard criteria.

PathogenWatch provided assembly metrics (i.e. genome size, contig count, N50 and GC content) and confirmed taxonomic confirmation via Mash-based comparison to RefSeq references (https://github.com/pathogenwatch-oss, accessed on 01 October 2025). In addition, *K. pneumoniae* genomes were assigned Life Identification Number (LIN) codes [92,93]. LIN codes are a hierarchical identifier derived from core-genome MLST similarity against Pasteur BIGSdb references (version 6.0.0-20250424); PathogenWatch returns a full or partial LIN plus the matched clonal group and sublineage, for context [12,14].

### Plasmid reconstruction and reference plasmid database construction

The software workflow ACCIO [94] is a bioinformatics tool for post-assembly identifying, clustering and tracking plasmids in bacterial genome assemblies using a custom reference plasmid database. ACCIO (create) was used to construct a local reference plasmid database using long-read assemblies as input. Plasmids were recovered as complete circular sequences >10 kb in length, which encoded plasmid replicons identifiable by blastn (≥80% similarity score) to the PlasmidFinder database or using MOB-typer. ACCIO’s deduplication steps were skipped; the database consisted of all plasmid sequences recovered from assemblies. The database was clustered into plasmid communities (coarse groupings indicating broad relatedness) and plasmid subcommunities (refined groupings indicating close relatedness, consistent with recent shared ancestry and/or putative recent horizontal transfer). A conservative containment distance threshold of <0.3 (defines the same community) and DCJ-Indel distance of ≤4 (defines the same subcommunity in addition to containment <0.3) were used for Pling clustering parameters to connect plasmids that are similar enough to represent possible recent transmission events [95]. For each isolate assembly, ACCIO (find) was used to detect the presence of database plasmids and plasmid subcommunities from the assembled contigs. In the same step, isolates were also screened for putative unresolved plasmids through the identification of leftover plasmidic contigs (PLASMe), which did not match any database plasmid. Unmatched plasmidic contigs were used for plasmid reconstruction with MOB-recon.

### Whole-chromosome variant calling and SNV density workflow

Quality-trimmed and filtered nanopore reads from the *K. pneumoniae* genomes were aligned to their respective reference chromosomes with minimap2 (configured with the ‘map-ont’ preset). The resulting Sequence Alignment/Map (SAM) files were converted to Binary Alignment/Map (BAM) format, then sorted and indexed with SAMtools v1.20 [96]. Mean sequencing depth was calculated using the ‘depth’ function in SAMtools, with the ‘-aa’ flag and a minimum mapping quality of 1. Per-base depths were averaged within 1,000 bp windows using an AWK command and sorted by genome coordinate:


$$\begin{array}{r} \text{samtools depth -aa -q 1 \$\{SAMPLE\}.sorted.bam |}\\ \;\text{awk `\{}\\ \;\text{bin=int(\$2/1000)*1000;}\\ \;\text{cov[bin]+=\$3;}\\ \;\text{n[bin]++;}\\ \;\text{\}}\\ \;\text{END\{}\\ \;\text{for(b in cov) print b, cov[b]/n[b];}\\ \;\text{\}` |}\\ \;\text{sort -n} \end{array}$$

Variants were called with Clair3 v1.0.8 [97] using a model matched to the nanopore chemistry (i.e. ‘r941_prom_sup_g5014’ for R9.4.1) and flags ‘--include_all_ctgs’, ‘--no_phasing_for_fa’, and ‘--haploid_precise’. Variant calls were filtered using BCFtools v1.20 [98], retaining SNVs with a Phred-scaled quality score ≥20 (equivalent to an expected error rate of one incorrect base call per 100 observations). Genome-wide SNV densities were visualized in R v4.2.2 [99] using the ggplot2 v3.4.0 package [100], by binning filtered SNV positions into 1,000 bp windows across each reference and plotting bar heights as counts per bin (integer *y*-axis).

### Global contextual analysis and phylogenetic reconstruction

To contextualize our genomes with publicly available data, *K. pneumoniae* genomes were identified using the PathogenWatch platform. Corresponding paired-end sequence read data (FASTQ files) were retrieved from the NCBI SRA using the ‘fasterq-dump’ utility within the SRA Toolkit v3.0.1-ubuntu64 (https://github.com/ncbi/sra-tools, accessed on 14 April 2025), for downstream analyses. SNVs were identified using the SPANDx v4.0.4 pipeline [101], which performs read mapping and variant calling across multiple genomes. Illumina short reads or simulated short reads generated from complete nanopore assemblies (see SPANDx documentation) were aligned to a closed reference chromosome. Variant positions within dense clusters (≥3 SNVs per 10 bp), mobile genetic elements or predicted regions of recombination were excluded from the core-genome alignment. Recombination was inferred using Gubbins v3.3.5 [102] on the pseudochromosome multiple-sequence alignment generated by projecting reference-mapped SNVs onto the reference backbone to preserve genomic coordinates. Additional filtering removed sites with sequencing depths <0.5× or >3× the mean coverage for each genome. The core genome was defined as regions (100 bp windows) present at ≥95% breadth of coverage across all isolates, determined using the BEDTools v2.28.0 [103] coverageBed module within the SPANDx pipeline. The resulting sets of non-recombinant core-genome SNVs were used to reconstruct a maximum-parsimony phylogeny in PAUP v4.0a [104], using the heuristic search algorithm with 1,000 bootstrap replicates to assess node support. Pairwise SNV distances between genomes were calculated using snp-dist v0.6.3 (https://github.com/tseemann/snp-dists, accessed on 11 November 2024). Phylogenetic trees were visualized using FigTree v1.4.4 (http://tree.bio.ed.ac.uk/software/figtree/, accessed on 11 November 2024).

## Results

### Older adults are disproportionately represented in this patient cohort, with nearly half over 65 years old

From January 2022 to April 2023, 157 *K*. *pneumoniae* isolates were sequenced onsite at Wellington Regional Hospital (Tables 1 and S1), representing 152 patients. Multiple isolates were obtained from four patients (three isolates from one patient and two isolates each from three patients). The median patient age was 64 years [interquartile range (IQR): 39 to 77], and 82/152 (54.0%) were female (the remainder male, 70/152, 46.0%). Isolates were frequently recovered from patients aged ≥65 years (*n*=75/152, 49.3%), with adults aged 45 to <65 years contributing a further 23.0% (*n*=35/152). Younger age groups were less represented, including 10 isolates (6.6%) from children under 5 years and four isolates (2.6%) from those aged five to <18 years. Urine was the predominant specimen (94/157, 60.3%), followed by blood cultures (21/157, 13.4%), respiratory samples (11/157, 7.0%), swabs (10/157, 6.4%), aspirates (9/157, 5.7%) and tissue (4/157, 2.5%). Screening samples comprised only 7/157 isolates (4.5%), consistent with targeted, risk-based screening rather than routine or universal testing. At the time of sampling, 94/152 (61.8%) patients were inpatients, 52/152 (34.2%) were seen in the Emergency Department or Outpatient clinics and 6/152 (3.9%) were Emergency Department/Outpatients with an inpatient admission in the prior 90 days. Using a 72-h threshold relative to hospital admission, 122/157 (77.7%) samples were community-onset and 35/157 (22.3%) hospital-onset (Table 1, predominantly urine and respiratory specimens). Phenotypic antimicrobial susceptibility testing indicated high susceptibility overall (meropenem 98.6%, ceftriaxone 89.6%, gentamicin 92.2% and ciprofloxacin 86.8%) with an ESBL phenotype detected in 7.1% of isolates (Table S2).

**Table 1.** Baseline patient demographics and sampling context (specimen type, location and timing) for *K. pneumoniae* isolates at Wellington Regional Hospital, January 2022 to April 2023

Characteristic	Timing of inpatient sample collection	
	Pre-72 h	Post-72 h
Age (years), median (interquartile range)	63 (39 to 77)	67 (35 to 75)
Female	65 (53.8)	19 (55.9)
Sample type		
Urine	77 (65.3)	16 (47.1)
Blood culture	18 (15.3)	3 (8.8)
Swab	8 (6.8)	2 (5.9)
Respiratory sample	3 (2.5)	7 (20.6)
Aspirate	3 (2.5)	4 (11.8)
Screening sample	6 (5.1)	1 (2.9)
Tissue	3 (2.5)	1 (2.9)
Patient location at time of sample collection		
Inpatient	61 (51.7)	34 (100.0)
Emergency Department or Outpatients	51 (43.2)	0 (0)
Emergency Department or Outpatients with inpatient admission in the prior 90 days	6 (5.1)	0 (0)

Values are *n* (%) unless stated; age shown as median (IQR).

#### 121 high-quality genomes obtained after quality control

A total of 36 genomes had a genome depth <25× and were excluded from further analyses (Table S3). Following quality control filtering, 121 genomes (77%) were retained for downstream analyses, with genome-level assembly and quality metrics summarized in Table S4. Median read depth was 64-fold (IQR: 40× to 87×; range 25× to 277×), with a median read N50 of 22.4 kb (IQR: 19.3 to 25.3 kb; range 11.0 to 33.2 kb). Genome assemblies had a median length of 5.5 Mb (IQR: 5.4 to 5.8 Mb; range 5.1 to 6.4 Mb) and a median of three contigs (IQR: 2 to 4; range 1 to 27), consistent with the expected *K. pneumoniae* genome size and structure. Of the 121 genomes, 118 were circularized and complete, while 3 remained as high-quality drafts.

Across the 118 complete genomes (228 total plasmids with an identified replicon type), the median plasmid count was 2 (IQR: 3 to 1; range 0 to 12) per genome; with a median plasmid size of 74.0 kb (IQR: 5.4 to 170.3 kb; range 2.3 to 357.9 kb). Plasmid counts did not differ between predicted ESBL-producing (*n*=11) and non-ESBL strains (*n*=107) (median 2 vs. 2; Mann–Whitney *U*=519.5, *P*=0.97) or between multidrug-resistant (*n*=18) and non-multidrug-resistant strains (*n*=100) (median 2 vs. 2; Mann–Whitney *U*=864.5, *P*=0.86).

### The local *K. pneumoniae* population was highly diverse

**Fig. 2.**
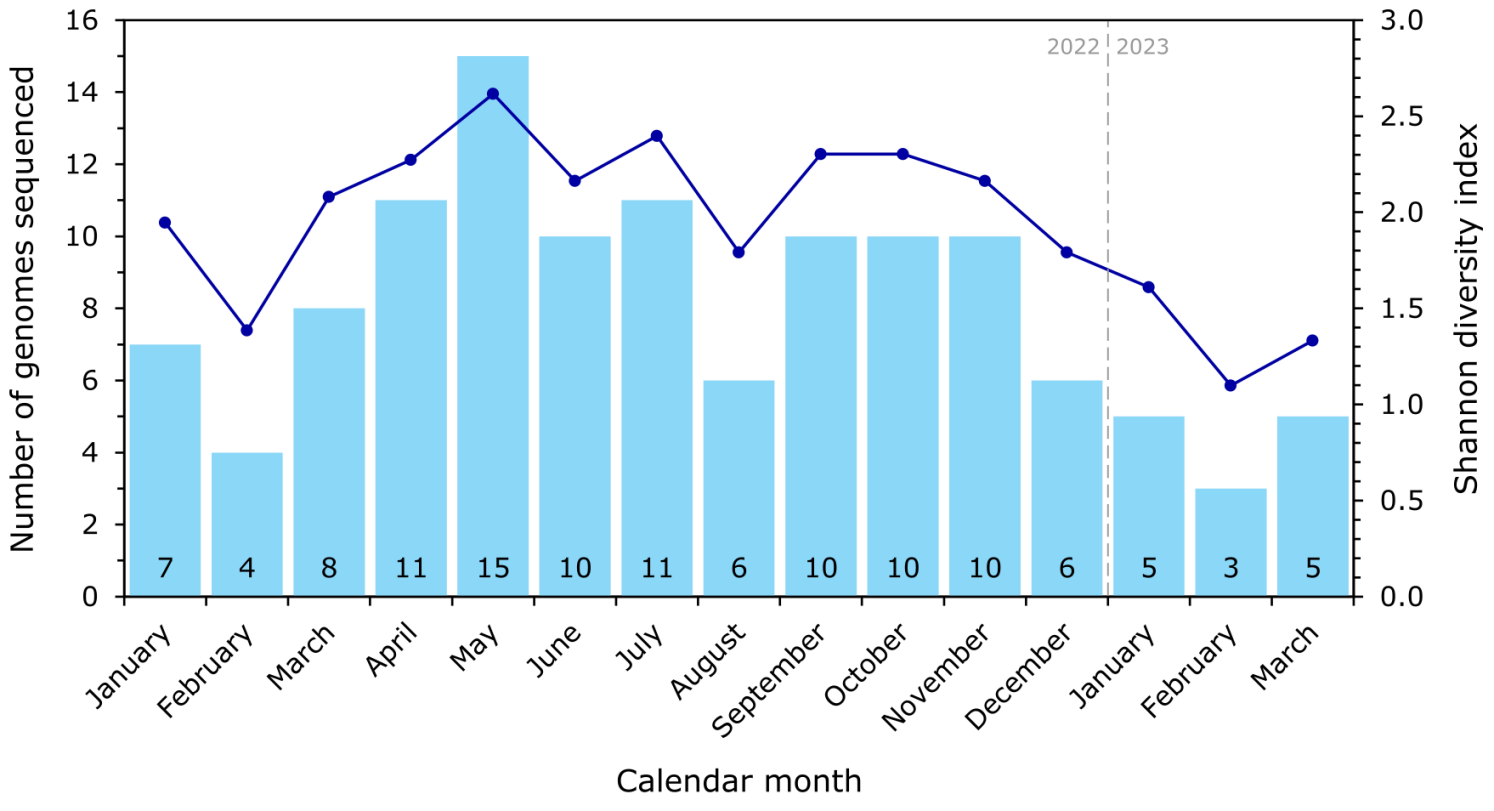
Monthly sequencing counts and diversity of *K. pneumoniae* sequence types at Wellington Regional Hospital. Blue bars indicate the number of genomes sequenced each month (range 3 to 15; totals shown at the base of each bar). The dark blue line shows the Shannon diversity index, which remained consistently high throughout 2022 (≥2.0 in most months), despite fluctuations in sequencing volume, and declined modestly in early 2023 when fewer isolates were sequenced.

**Fig. 3.**
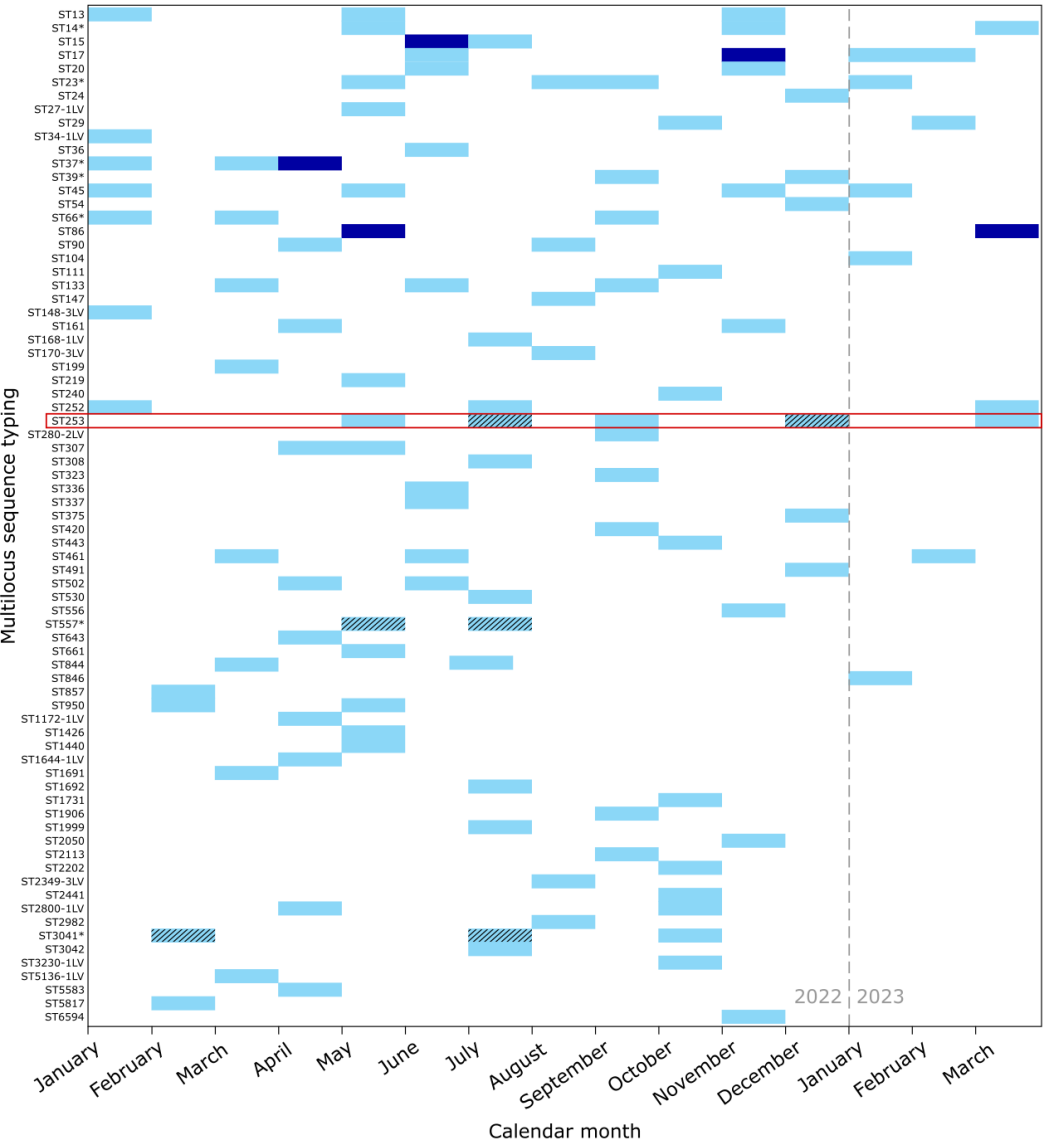
Monthly counts of *K. pneumoniae* lineages at Wellington Regional Hospital. The heatmap shows genome counts per MLST by calendar month for 121 genomes sequenced. Shading indicates the number of genomes (light blue, 1; dark blue, 2). Black diagonal hatching indicates genome pairs separated by <50 core-genome single-nucleotide variants. ST labels marked with an asterisk (*) denote groups that include the parent ST together with closely related single-locus or double-locus variants (e.g. ST14 and ST14-1LV). The most common lineage was ST253 (highlighted in red), which recurred intermittently across 5 months.

A total of 75 distinct STs were identified. Despite fluctuations in sequencing volume (median eight genomes/month, range 3 to 15), ST diversity remained consistently high throughout 2022 (Shannon indices ≥2.0 in most months), peaking at 2.62 in May 2022 when the largest number of genomes (*n*=15) were sequenced (Fig. 2). Although ST diversity declined slightly in early 2023 alongside lower numbers of genomes sequenced (Shannon index 1.10 to 1.61), multiple STs continued to co-circulate, indicating a persistently heterogeneous population. Most STs (51, 68%) were detected only once (Fig. 3), with a small number observed repeatedly across multiple months, consistent with a predominantly sporadic population structure rather than sustained clonal expansion.

Predicted surface polysaccharide diversity was similarly high, with 55 distinct K loci identified across 118/121 genomes and 13 distinct O types predicted (Table S4). No single K locus dominated, and most were represented by only one or two isolates (*n*=39, 71% of K loci), although KL2 (*n*=9) and several others (KL116, KL24, KL10 and KL15; *n*=5 each) were more frequently observed. K loci in 95 genomes were reported with gene truncations predicted to result in the capsule null phenotype; however, such truncations should be interpreted with caution, given known homopolymer-associated sequencing errors in genomes assembled solely using Oxford Nanopore R9.4 flow cell sequencing data [45]. In contrast, O-antigen diversity was more skewed, with O1/O2 variants predominating (67.8% of genomes), while other O types were detected at lower frequency (Table S4).

Acquired AMR and virulence determinants were present in a minority of genomes, but were themselves diverse. Twenty-seven genomes (22%) carried acquired AMR genes (median 5, range 1–18), including 11 with ESBLs and a single genome harbouring a carbapenemase gene (*bla*_NDM-1_). The most common ESBL gene was *bla*_CTX-M-15_ (*n*=8 genomes, one with two copies), but two genomes each carried two copies of *bla*SHV-12 and one genome carried *bla*_CTX-M-3_. Virulence loci were also variably distributed: 37 genomes (31%) carried the yersiniabactin siderophore locus across multiple chromosomal integrative conjugative element (ICE)Kp variants, including seven with ICEKp10 (colibactin-positive). The majority of *ybt* loci and all *clb* loci were reported as truncated (Table S5), likely reflecting sequencing and/or assembly artefacts, although true biological variation cannot be excluded. Aerobactin (*iuc*) was detected in 17 genomes (14%), typically alongside *rmp*ADC and salmochelin (*iro*) loci, consistent with known virulence plasmid architectures [105,106].

### Limited acquisition of AMR determinants

All genomes carried intrinsic chromosomal resistance determinants typical of *K. pneumoniae*, including *bla*_SHV_ alleles (predominantly *bla*_SHV-1_ and *bla*_SHV-11_), along with *fos*A, *oqx*AB and *emr*D (Table S6). Just four completed genomes carried acquired AMR genes on the chromosome (*n*=2 ST15, *n*=1 ST147 and *n*=1 ST219). In two ST15 co-isolates from distinct colonies derived from a single clinical specimen, complete assemblies resolved a chromosomal IS*26*-Tn*3* composite module inserted between *pla*P (putrescine/proton symporter) and *fus*A (elongation factor G). The module carries a fixed cassette (*qnr*B1-*bla*_SHV-12_-*aac*(6′)-*Ib-cr5-bla*_OXA-1_-*cat*B3) within IS*26*-Tn*3* architecture (including Tn*5403*) arranged as head-to-tail tandems: three copies in kp220713_barcode45 and two in kp220713_barcode46 (Fig. S1). In both genomes, *aac*(3)-*IIe* sits immediately downstream of the distal end of the tandem array. Following read mapping of kp220713_barcode46 (two repeats) to kp220713_barcode45 (three repeats), Clair3 identified a single high-confidence synonymous SNV outside mobile elements and predicted recombination [position 1,109,007 relative to kp220713_barcode45, i.e. A364G (Leu122Leu)] within *jef*A (encoding a drug-efflux gene). Phenotypically (Vitek^®^ 2 S/I/R), kp220713_barcode45 (three copies) showed shifts toward non-susceptibility for selected oxyimino-beta-lactams compared to kp220713_barcode46 (two copies). These included cefepime (category step values ~≥64 non-susceptible; ~2 intermediate) and ceftriaxone (≥64 vs. ~16; both non-susceptible). Both isolates were non-susceptible to ceftazidime (≥64). Additional differences were observed for cefoxitin (kp220713_barcode45 non-susceptible ≥64; kp220713_barcode46 susceptible ≤4), while meropenem remained susceptible in both (kp220713_barcode45~2; kp220713_barcode46 ≤0.25). These values are Vitek^®^ 2 category step values and not reference broth-microdilution MICs.

In kp220906_barcode90 (ST147), *bla*_CTX-M-15_ was chromosomally integrated (position 3,877,541 to 3,878,413 bp; GenBank: JBREMR000000000). The *bla*_CTX-M-15_ gene was embedded within an IS*Ecp1-bla*_CTX-M-15_ transposition unit. In kp220531_barcode70 (ST219), two chromosomal copies of *bla*_CTX-M-15_ were present at positions 206,126–206,998 bp and 1,166,725–1,167,597 bp (GenBank: JBRENY000000000). Both copies were associated with upstream IS*Ecp1* (IS*1380* family transposase), indicating independent mobilization via the IS*Ecp1*-mediated mechanism and multiple insertion events rather than tandem duplication within a single locus.

### No substantive evidence for nosocomial transmission

We used whole-genome SNV counts to investigate putative nosocomial transmission events within our hospital. There is an emerging consensus that pairs of isolates differing by ≤20 to 25 SNVs reflect likely transmission events [45,107, 108] when the data are derived from Illumina whole genome sequences, while a relaxed threshold of <50 SNVs was recently proposed for data generated using the Oxford Nanopore R9.4 flow cell, as generated here [45]. We, therefore, counted SNVs among all genome pairs that represented the same core-genome sequence type (cgST) (Figs S3, S4, S5, S6, S7, S8 and S9). Among the ten pairs of genomes representing the same cgST, there were three that differed by <50 SNVs. The closest pair [closest cgST30572, 7-gene ST253, differing by three filtered SNVs; kp220713_barcode53 (GenBank: JBRENG000000000) and kp221229_barcode17 (GenBank: JBRELJ000000000)] was collected from the same patient sampled five months apart, consistent with within-host persistence and microevolution rather than transmission. The next closest pair (closest cgST5879, 7-gene ST557, genomes kp220517_barcode26, GenBank: JBREOB000000000; kp220809_barcode36, GenBank: JBREMY000000000) differed by 12 filtered SNVs. Although this falls near the lower bound for possible relatedness, the isolates originated from different and unrelated patients (May 2022 vs. July 2022, respectively) with no temporal or known clinical overlap. Although environmental sampling was not performed and rectal screening was limited, the available clinical and screening isolates spanned multiple wards, months and patient groups with no shared epidemiological links. Read mapping for the final pair (closest cgST12105, 7-gene ST3041, kp220309_barcode93 and kp220726_barcode01, differing by 32 filtered SNVs) identified four regions present in kp220726_barcode01 that were absent from kp220309_barcode93, in addition to the observed SNVs. These structural differences were considered alongside the SNV distance when assessing relatedness and do not support a recent transmission event. Across all genome pairs, most differed by>50 pairwise SNVs, and SNVs were distributed across the genome rather than clustered (Figs S3, S4, S5, S6, S7, S8 and S9). Combined with the observed ST diversity and the lack of epidemiological links, these data do not support sustained clonal transmission within Wellington Regional Hospital during the study period.

**Fig. 4.**
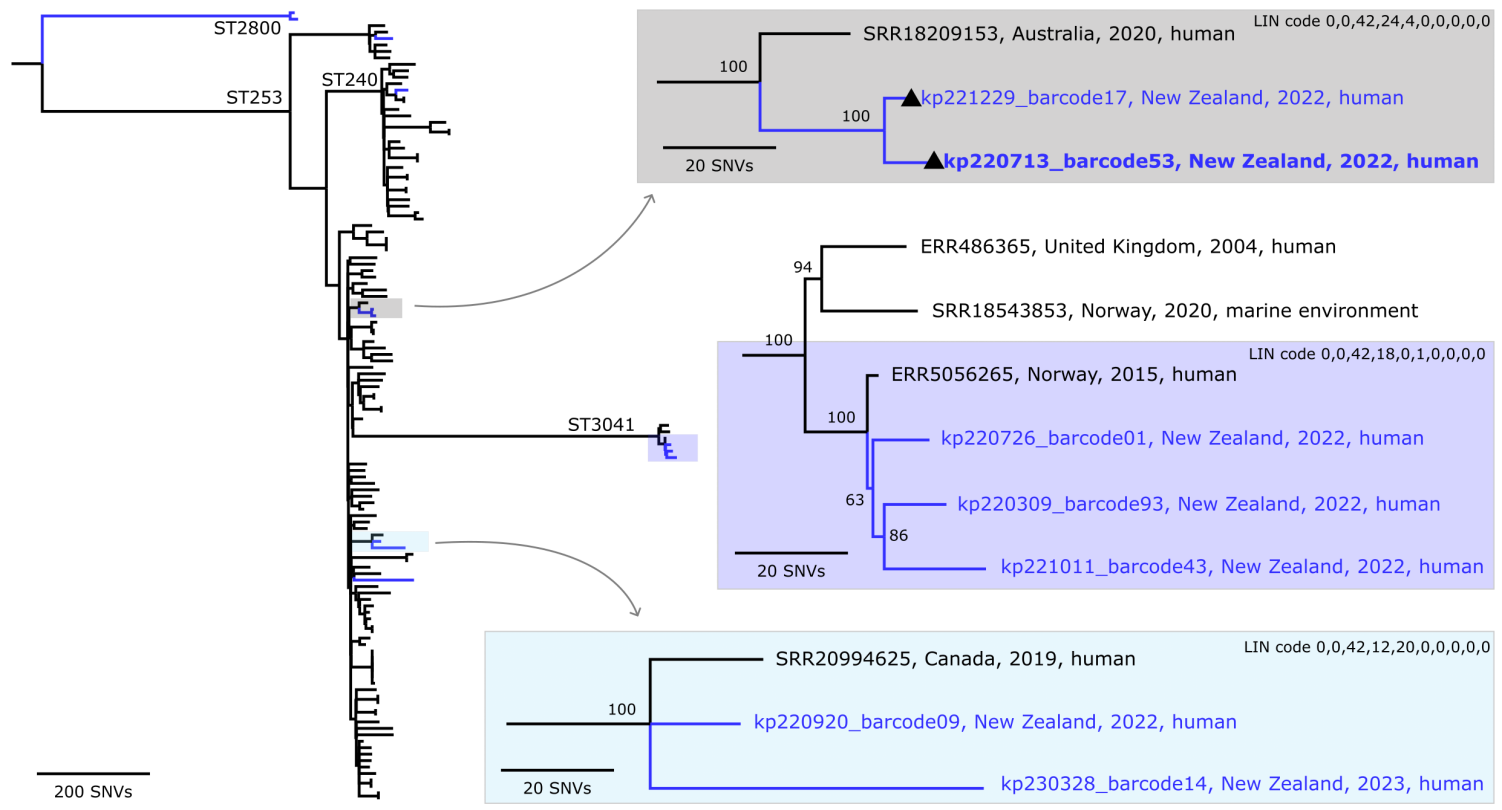
Maximum-parsimony phylogeny of the *K. pneumoniae* ST253 and closely related genomes. The phylogeny was inferred from 5342 non-recombinant orthologous biallelic core-genome SNVs from 123 genomes. SNVs were derived from a core-genome alignment of ~4,160,100 bp and were called against the chromosome of kp220713_barcode53 (GenBank: JBRENG000000000). The consistency index for the tree was 0.99. SNV density filtering in SPANDx (excluded regions with three or more SNVs in a 10-bp window). The phylogenetic tree was rooted to the ST2800 outgroup. Branch colours represent genomes from the public domain (black) and this study (blue). Numbers (right plots) represent bootstrap values (1,000 replicates) for that node. (▲) Isolates kp220713_barcode53 (July 2022) and kp221229_barcode17 (December 2022) were collected from the same patient.

**Fig. 5.**
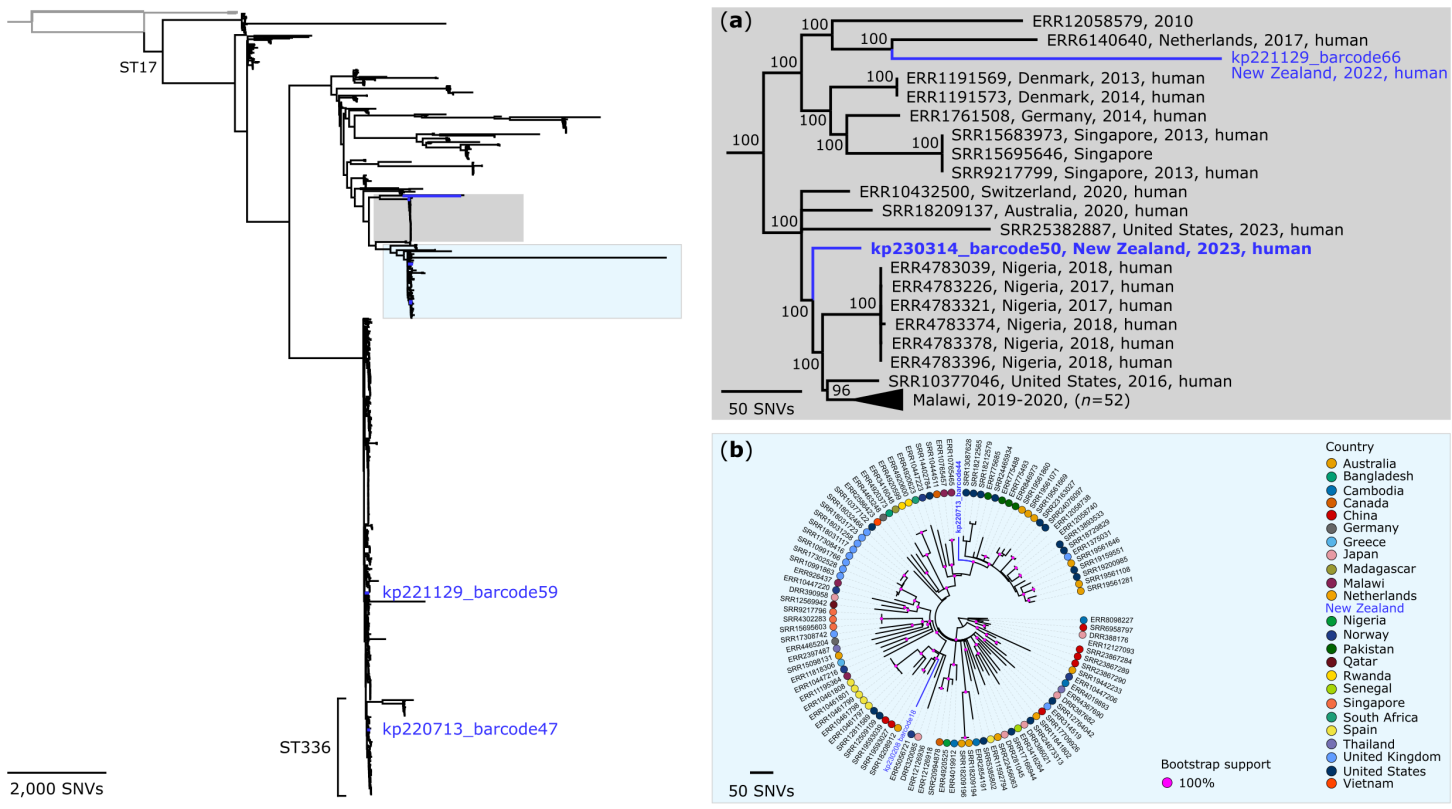
Maximum-parsimony phylogeny of the *K. pneumoniae* ST17 genomes. (**a**) The phylogeny was inferred from 1,117 non-recombinant orthologous biallelic core-genome SNVs from 72 genomes. SNVs were derived from a core-genome alignment of ~4,888,400 bp and were called against the chromosome of kp230314_barcode50 (GenBank: JBREKZ000000000, LIN code: 0,0,22,12,75,0,0,0,0,0). (**b**) The phylogeny was inferred from 5,125 non-recombinant orthologous biallelic core-genome SNVs from 104 genomes. SNVs were derived from a core-genome alignment of ~4,622,300 bp and were called against the chromosome of kp220713_barcode44 (GenBank: JBRENK000000000, LIN code: 0,0,22,12,12,3,0,0,0,0). The consistency index for both trees was 1.0. SNV density filtering in SPANDx (excluded regions with three or more SNVs in a 10-bp window). The phylogenetic trees were rooted to the ST17 lineage structure (left). The ST17 assembly-based phylogeny (broader context for **a **and **b**) was inferred from 66,317 core-genome SNVs from 1,105 genomes. SNVs were derived from a core-genome alignment of ~3,424,230 bp and were called against the chromosome of kp220713_barcode44. Branch colours represent genomes from the public domain (black) and this study (blue). Numbers (**a**) or pink circles (**b**) represent bootstrap values (1,000 replicates) for that node.

To place the predominant lineages into a broader context, we compared the local genomes with publicly available representatives from related lineages. The ST253 and ST17 genomes from Wellington Regional Hospital were interspersed among publicly available global genomes (Figs4  5). Small clusters of genomes were observed, with pairwise SNV distances ranging from 12 to 75 SNVs for ST253, excluding mobile genetic elements and regions of recombination (Tables S7 and S8). As mentioned, the kp220713_barcode53 and kp221229_barcode17 represent two isolates from the same patient sampled months apart (July and December 2022). Excluding this longitudinal pair, the compared isolates in Figs 4 and 5 originated from different wards, time periods (July 2022 to March 2023) and patient groups, with no shared clinical or epidemiological link (Table S1).

#### Globally distributed clones were present, but were not particularly problematic in our setting

Several globally distributed infection-associated clones were identified, including hypervirulence-associated ST23 (*n*=3), ST66 (*n*=2), ST86 (*n*=4) and ST375 (*n*=1, single-locus variant of ST65). In all cases, the genomes carried the canonical K loci associated with these hypervirulent clones (KL1 for ST23, KL2 for the remainder), as well as each of the virulence plasmid-associated *iuc*, *iro* and *rmpADC* loci. These lineages presented with both hospital- and community-onset infections across varied clinical sites. ST23 was detected in community-onset cases involving a Bartholin’s abscess and urine and in a hospital-onset sputum isolate. ST66 occurred in a hospital-onset sternomastoid abscess aspirate and a community-onset bloodstream infection. ST86 was restricted to community-onset disease, spanning two urinary isolates, one bloodstream infection and one liver aspirate. ST375 was identified in a hospital-onset sputum isolate (Tables S1 and S4). Several presentations included liver and deep-tissue abscesses and bloodstream infection.

Globally distributed multidrug resistance-associated clones included ST14 (*n*=3), ST15 (*n*=3), ST17 (*n*=5), ST20 (*n*=2), ST29 (*n*=2), ST37 (*n*=4), ST147 (*n*=1) and ST307 (*n*=2). These lineages caused community-type presentations [predominantly urinary isolates (ST14, ST15, ST17, ST20, ST29, ST37, ST307), together with soft-tissue or wound infections (ST17, ST20, ST29) and one bloodstream infection (ST14)]. No evidence of nosocomial transmission was observed for any of the globally distributed multidrug-resistant clones, and only a subset of the isolates carried genetic determinants of multidrug resistance (defined as acquired resistance to ≥3 drug classes). Multidrug-resistant determinants were detected in one ST14 carrying the *bla*_CTX-M-3_ ESBL gene plus resistance determinants for eight additional antimicrobial classes; two ST15 each carrying two copies of the *bla*_SHV-12_ ESBL gene plus determinants for three additional classes; two ST37 isolates with resistance determinants for four and eight classes, respectively (no ESBL or carbapenemases); one ST147 isolate carrying *bla*_CTX-M-15_ plus determinants to six other classes; and two ST307 each carrying the *bla*_CTX-M-15_ ESBL gene plus determinants for six and seven additional classes, respectively. These isolates were identified across multiple clinical presentations, predominantly community-onset, with no epidemiological links identified between cases.

### Plasmids as the primary reservoir of acquired AMR and virulence

Our data provided an opportunity to explore plasmid content across an entire clinical isolate collection and confirm the relative roles of chromosomes and plasmids in the acquisition of AMR and virulence genes. As described above, plasmids were common across the dataset, with most genomes carrying one or more plasmids with no association between plasmid burden and resistance phenotype. We, therefore, focused on plasmid gene content and distribution of these plasmids to determine their contribution to AMR and virulence.

Plasmid reconstructions spanned diverse incompatibility groups but were dominated by Col, F-type and multi-replicon elements (Fig. S10). Col plasmids (*n*=54) were consistently small (<15 kb), while IncR plasmids (*n*=5) showed intermediate sizes (median: 82.8 kb). F-type plasmids were the most frequent (*n*=109) and displayed substantial size variation (median, 148.5 kb; IQR, 107.6 to 194.4 kb). Multi-replicon plasmids (>1 distinct Incompatibility group) were also common (*n*=24) and tended to be large (median: 215.9 kb).

#### Plasmid population structure reveals limited clustering of closely related elements

**Fig. 6.**
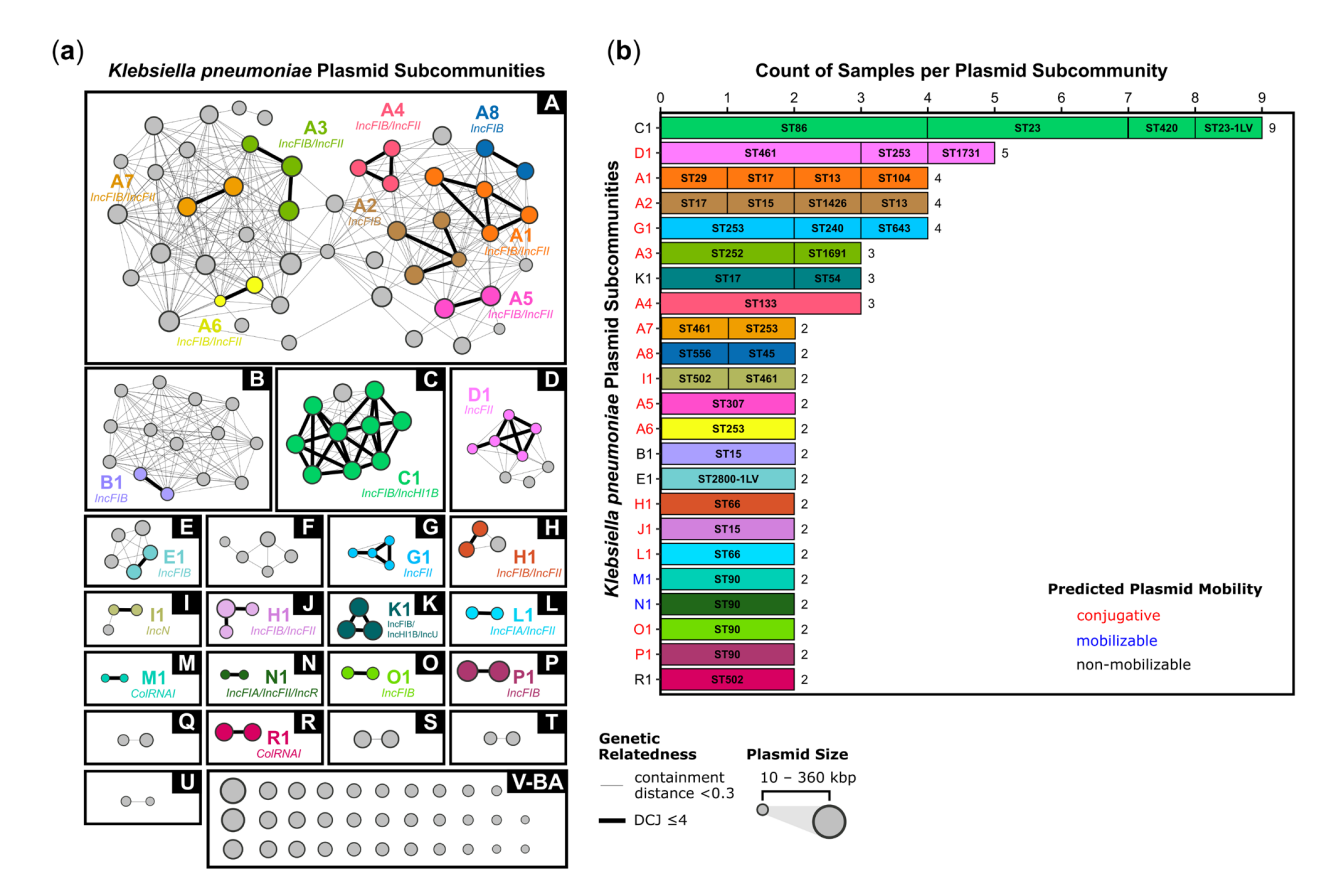
Plasmid relatedness and host distribution of *K. pneumoniae* plasmid subcommunities. (**a**) The local *K. pneumoniae* reference plasmid database (*n*=158 plasmids, scaled by length ~10–360 kb) was constructed using ACCIO; Pling was used to infer plasmid relatedness and define plasmid communities (boxed; labelled A-BA by community size) based on a containment distance threshold of <0.3. Within each community, subcommunities (coloured groups; labelled community letter plus subcommunity number for subcommunities containing ≥2 plasmids) represent closely related plasmids defined by high structural similarity using a DCJ–indel distance threshold of ≤4 for plasmids <120 kb, +1 DCJ per additional 20 kb. Light grey edges indicate plasmid pairs meeting the containment distance threshold; black edges indicate plasmid pairs meeting both the containment distance and DCJ–indel thresholds for relatedness. Light grey nodes represent plasmids that lacked strong relatedness to any other plasmid (assigned a unique subcommunity). (**b**) Counts of plasmid subcommunity calls across 121 *K*. *pneumoniae* isolate assemblies are shown for subcommunities containing >1 plasmid, obtained by screening each assembly with ACCIO against the local reference plasmid database. Horizontal bars are partitioned by host MLST. The total number of isolates with each subcommunity call is listed. Predicted plasmid mobility for each subcommunity is indicated by label colour on the *y*-axis (red, conjugative; blue, mobilizable; black, non-mobilizable).

A local plasmid database was constructed from 121 *K*. *pneumoniae* long-read assemblies using ACCIO (Fig. 6a, Table S9). The database consisted of 158 plasmids (p1-p158 numbered chronologically within each subcommunity; length >10 kb, range ~10–357 kb, median length 139 kb), which were clustered into 53 communities (A-BA by descending size; range 1–48 plasmids) (Table S10). Among the 21 plasmid communities, which contained ≥2 plasmids (39.6%), the median community size was 3 plasmids.

Within these communities, the database was further divided into 115 subcommunities, which represented groups of closely related plasmids (A1-BA1 by descending size within community), including 92 singleton subcommunities containing only 1 plasmid (80.0%) and 23 subcommunities with ≥2 plasmids (20.0%, median size *n*=2, range 2–9 plasmids). The vast majority of plasmid subcommunities (*n*=97, 84.3%) contained 1 or more identifiable IncF-family replicons (i.e. IncFIA, IncFIB, IncFIC or IncFII), and 38 (33.0%) contained plasmids with one or more AMR genes (AMRfinder hits with >80% blastn similarity score) (Table S11). The largest community, Community A, consisted of 48 IncF family plasmids (all with IncFIB and/or IncFII replicons), which were further clustered into 34 subcommunities, 8 of which contained ≥2 plasmids.

#### Plasmid sharing across sequence types without evidence of local dissemination

The distributions of plasmid subcommunities with ≥2 plasmids across *K. pneumoniae* MLSTs are shown in Fig. 6b. Amongst the 23 total subcommunities with ≥2 plasmids, 10 (43.5%) were detected in more than one MLST, and 13 (56.5%) were detected in a single ST only. Of the subcommunities with ≥2 plasmids, a majority (*n*=16, 69.6%) were predicted to be conjugative, five (21.7%) non-mobilizable, and two (8.7%) mobilizable (MOB-suite).

The largest subcommunity, Subcommunity C1, contained nine highly similar IncFIB/IncHI1B plasmids predicted to be non-mobilizable and was observed across nine samples from four distinct MLSTs (ST86, ST23, ST420 and ST23-1LV). Nine other plasmid subcommunities were observed in ≥2 MLSTs, including Subcommunity D1 (IncFII; ST461, ST253, ST1731), A1 (IncFIB/IncFII; ST29, ST17, ST13, ST104), A2 (IncFIB; ST17, ST15, ST1426, ST13), G1 (IncFII; ST253, ST240, ST643), A3 (IncFIB/IncFII; ST252, ST1691), K1 (IncFIB/IncHI1B/IncU; ST17, ST54), A7 (IncFIB/IncFII; ST461, ST253), A8 (IncFIB; ST556, ST45) and I1 (IncN; ST502, ST461). Of the ten plasmid subcommunities observed in more than one MLST, eight were predicted to be conjugative, whereas two were predicted to be non-mobilizable.

#### Plasmid-encoded resistance determinants account for clinically relevant AMR

Plasmid-encoded AMR genes were identified in 35/118 genomes (29.7%) and were associated with a broader and more clinically relevant repertoire of resistance determinants than those found chromosomally (Table S12). Beta-lactam resistance genes occurred on plasmids in 19 genomes (16.1% of the total dataset), including *bla*_TEM-1_ (*n*=8 genomes), *bla*_CTX-M-15_ (*n*=6 genomes), *bla*_SHV-1_ (*n*=4 genomes), *bla*_DHA-1_ (*n*=3 genomes), *bla*_OXA-1_ (*n*=2 genomes) and single detections of *bla*_CTX-M-3_, *bla*_NDM-1_ and *bla*_OXA-10_.

Non-beta-lactam resistance determinants were also frequently detected on plasmids (*n*=35 genomes): trimethoprim resistance genes were detected in 30/35 genomes (85.7%), sulphonamide resistance in 18 (51.4%), quinolone resistance in 16 (45.7%), tetracycline efflux pumps in 15 (42.9%) and aminoglycoside-modifying enzymes in 14 (40.0%). Macrolide and phenicol resistance loci occurred in 7/35 genomes (20.0%), while rifampicin resistance was rare (*arr*-2 and *arr*-3, each in a single genome, 2.9%). The most frequent individual plasmid genes were *sul*1 and *tet*(A) (each in 12 genomes, 34.3%), *dfrA*50 (11, 31.4%), *dfr*A14 (10, 28.6%) and multiple quinolone resistance determinants, including *qnr*S1, *qnr*B1 and *mph*(A) (each in 6 genomes, 17.1%). Ten of the 13 completed genomes harbouring ESBL genes contained them on plasmids.

#### Resistance-sparse F-type plasmids predominate despite similarity to global multidrug resistance backbones

To assess whether shared AMR plasmids or conserved resistance architectures were circulating between strains, we examined representative F-type plasmids closely related to well-characterized global AMR plasmid backbones. These reference plasmids typically carry extensive multidrug-resistance regions, allowing evaluation of whether similar resistance modules or evidence of recent plasmid transmission were present within the hospital isolate collection. Across all comparisons, plasmids in this study shared backbone similarity with globally disseminated AMR plasmids but lacked their characteristic multidrug-resistance regions, instead carrying only a small subset of resistance genes.

The pkp22071345A plasmid (GenBank: JBRENJ000000000; MOB-suite cluster AA277) is a 221,335-bp conjugative F-type plasmid (IncFIB, IncFII) similar to pJYC03A (GenBank: CP022920; Mash 0.0248). While pJYC03A contains a complex IS*26-*associated multidrug-resistance region, including beta-lactam (*bla*_OXA-9_ and *bla*_TEM-1_, while *bla*_KPC-2_), aminoglycoside (APH(6)-Id), trimethoprim (*dfr*A14), tetracycline [*tet*(A)] and fluoroquinolone (*qnr*B1) resistance genes, pkp22071345A carries only *tet*(A) and *qnr*B1, with no genomic evidence of the broader multidrug-resistance module.

A similar pattern was observed for pkp22071346A plasmid (GenBank: JBRENI000000000; MOB-suite cluster AA458), a 114,126-bp non-mobilizable F-type plasmid (IncFIB) related to p160070-catA (GenBank: MG288676; Mash 0.0297). The reference p160070-catA plasmid carries a multidrug-resistance region comprising phenicol (*cat*A2), trimethoprim (*dfr*A12), aminoglycoside (*aad*A2, APH(3′)-Ia), sulphonamide (s*ul*1) and macrolide *[mph*(A)] resistance genes within IS*26*- and IS*6100*-flanked transposon modules. In contrast, pkp22071346A again carries only *tet*(A) and *qnr*B1, consistent with a resistance-sparse variant of the same IncFIB backbone.

Likewise, the pkp22071346C plasmid (GenBank: JBRENI000000000; MOB-suite cluster AA018), a 107,224-bp conjugative F-type plasmid (IncFIB, IncFII), is related to the carbapenemase-associated plasmid pG12-KPC-2 (GenBank: KU665642; Mash 0.0135). Whereas pG12-KPC-2 harbours *bla*_KPC-2_ within Tn*4401* alongside *bla*_OXA-9_, pkp22071346C carries only the *tet*(A) and *qnr*B1, with no evidence of carbapenemase-associated elements.

Finally, the pkp22090690A plasmid (GenBank: JBREMR000000000; MOB-suite cluster AA276) is a 188,240-bp conjugative F-type plasmid (IncFIB, IncFII) similar to pA1966-IMP (GenBank: MK036889; Mash 0.0168). pA1966-IMP contains an extensive IS*26*-rich multidrug resistance locus including beta-lactam (*bla*_IMP-4_, *bla*_TEM_, *bla*_SHV-1_ and *bla*_CTX-M-15_), tetracycline [*tet*(D)] and fluoroquinolone (*qnr*S1) AMR genes. In contrast, pKP22090690A contains only *dfrA*14. The presence of multiple insertion sequences in the reference backbone suggests IS-mediated rearrangement; however, the direction of gene gain or loss cannot be inferred.

Together, these comparisons indicate that although F-type plasmid backbones related to globally disseminated AMR plasmids are present in this collection, they predominantly occur as resistance-sparse variants. No conserved multidrug-resistance regions or shared AMR architectures were observed across strains, providing no evidence for recent horizontal transmission of high-risk AMR plasmids within the hospital. Consistent with chromosomal analyses, this supports a model in which plasmids contribute to resistance diversity without detectable nosocomial spread of multidrug-resistant plasmids (Fig. S11).

#### Evidence for additional unresolved plasmids

We assessed sample genomes for the presence of unresolved plasmids using ACCIO, i.e. plasmids that are not represented as a circular plasmid sequence in the database due to incomplete assembly. Of the 121 isolates, 50 (41.3%) were flagged by ACCIO as likely harbouring at least one additional plasmid. This indicates that, despite the high proportion of complete assemblies, some plasmid content remains unresolved within this dataset.

### Virulence determinants were limited and predominantly plasmid-encoded

Most accessory virulence determinants were plasmid-encoded, carried predominantly on F-type and multi-replicon plasmid backbones, with relatively few loci confined to the chromosome (Fig. S12). Across the complete genomes (*n*=118), yersiniabactin (encoded by *ybt*) was common, present in 37 genomes (31.4%). Almost all instances were chromosomally encoded (36/37, Table S13), with a single exception carried on a multi-replicon plasmid (strain kp221229_barcode16; IncFIB, IncHI1B, Table S14). These 37 *ybt*-positive genomes spanned diverse lineages, including ICEKp2, ICEKp3, ICEKp4, ICEKp5, ICEKp10, ICEKp11 and ICEKp12 variants, as well as one plasmid-borne ybt4. However, the majority were reported as truncated (Table S5).

In addition to canonical ICE-associated *ybt*, one genome (kp221229_barcode02; GenBank: JBRELN000000000) contained a large chromosomal region with high sequence similarity to a known virulence plasmid, including the aerobactin synthesis locus (*iuc*/*iut*), regulator *rmp*A2 and the salmochelin locus (*iro*BCDN). Comparative alignment revealed that this region is syntenic with a previously described virulence plasmid (2021CK-00720; GenBank: CP092537) but is integrated into the chromosome and flanked by multiple insertion sequences, consistent with plasmid-to-chromosome integration mediated by mobile genetic elements (Fig. S13). Colibactin (encoded by *clb*) was detected in seven genomes (chromosomally encoded), assigned to *clb* lineages one (*n*=2), two (*n*=2) and three (*n*=3), but all were flagged as truncated, indicating a predicted non-functional locus.

## Discussion

We have reported a comprehensive *K. pneumoniae* isolate collection, representing all clinical and screening isolates collected by the Wellington Regional Hospital clinical microbiology laboratory over a 15-month period. Most of the infection and colonization episodes reported during this time were classified as community – rather than hospital-onset (78.2% vs 21.8%, respectively) and comprised primarily urinary tract (60.3%), followed by bloodstream infections (13.5%). This approach prioritizes specificity over sensitivity; however, in the absence of systematic admission screening, some infections classified as hospital-onset may still represent community-acquired organisms detected later during admission. Aside from the expected intrinsic resistance to ampicillin, most isolates remained susceptible to clinically relevant antimicrobials, including meropenem (98.6% susceptible), ceftriaxone (89.6%), gentamicin (92.2%) and ciprofloxacin (86.8%), highlighting this as a comparatively low AMR burden setting [18]. This contrasts with international settings, where *K. pneumoniae* is frequently associated with multidrug resistance and carbapenemase production [2]. In this context, the combination of low AMR prevalence, high genomic diversity and the absence of sustained transmission is consistent with infections being largely driven by repeated, independent introductions rather than expansion of hospital-adapted clones. Our genomic data showed the *K. pneumoniae* population was highly heterogeneous, in terms of 7-gene STs, capsule and O antigens, as well as plasmids and virulence-associated mobile genetic elements. Notably, there was only sporadic recurrence of a few STs and no evidence of sustained in-hospital transmission (Figs2  3).

Within the limits of our sampling (clinical isolates and routine patient screening only), we found no genomically supported evidence of sustained in-hospital transmission at Wellington Regional Hospital during the study period. The clinical context supports this interpretation (sampling early in the care pathway, Table 1), as does high phenotypic susceptibility with only a small ESBL fraction (Table S2). Practically, this positions Wellington Regional Hospital in a non-endemic state where the priority for genomic analysis is rapid detection of instances of direct transmission. A trigger-ready genomic workflow is appropriate: in a non-endemic setting, routine onsite WGS can run light, with escalation only when predefined signals occur: (i) ≤20 to 25 core-genome SNVs on recombination-masked alignments plus epidemiologic linkage, (ii) re-appearance of the same ST within≤30 days in a unit, (iii) detection of high-risk plasmid backbones (e.g. IncL/M *bla*_OXA-48_-like) and (iv) a sustained rise in ESBL above local baseline. These triggers prompt targeted screening, infection-prevention review and intensified sequencing, while avoiding broad untargeted interventions.

In practice, routine sequencing can continue to be performed in pooled batches for efficiency; however, when a trigger is met, sequencing would shift to a rapid, targeted mode in which a small number of epidemiologically linked isolates (typically a handful of temporally or clinically related cases) are prioritized for immediate analysis rather than awaiting batch completion, reflecting the flexibility of decentralized sequencing models where routine batching can be interrupted to enable rapid outbreak investigation [22]. This strategy preserves resources, provides timely negative assurance when clusters are suspected and concentrates infection-prevention efforts on admissions and known high-risk events. Limitations remain (see the ‘Study limitations and strengths’ section), but the convergent genomic and clinical signals indicate no evidence of sustained nosocomial *K. pneumoniae* transmission at Wellington Regional Hospital during the study period (noting that environmental/carriage surveys were not performed).

Beyond the local diversity, these results highlight how genomic surveillance can complement infection-prevention practice in low-burden settings. Sequencing was embedded within the diagnostic workflow, and results were communicated to IPC clinicians in near real time, providing ongoing situational awareness of *K. pneumoniae* population structure within the hospital. No genomic clusters consistent with recent patient-to-patient transmission were identified, and therefore, no targeted IPC interventions were triggered for this organism during the study period. Nonetheless, the absence of detected transmission provided reassurance that existing infection-control practices were effective and that escalation was not required. In this context, the absence of detectable transmission had direct implications for local IPC practice. Specifically, the absence of detectable transmission among ESBL-producing *K. pneumoniae* isolates supported consideration of a move away from routine transmission-based precautions for these organisms, including single-room isolation, enabling more targeted use of limited isolation capacity for pathogens with clearer evidence of nosocomial spread. More broadly, these findings illustrate that the value of prospective genomic surveillance is not limited to outbreak detection. In low-transmission settings such as this, sequencing can provide evidence to safely de-escalate control measures, refine local risk assessments and inform resource allocation. As such, the utility of WGS extends beyond identifying transmission events to also demonstrating when transmission is not occurring.

Embedding sequencing directly within clinical laboratories [13,109] enables this kind of real-time assessment, supporting early detection when transmission does occur and allowing a shift from reactive outbreak management to proactive ‘stamp-it-out’ containment [22]. When integrated with national surveillance [57], distributed sequencing complements centralized reference services by delivering earlier, context-specific insight and generating data that are immediately actionable for IPC teams [14,22, 45]. This approach reflects New Zealand’s evolving direction for science (i.e. building collaborative, high-impact, high-value systems that translate research into tangible public-health benefits) while strengthening preparedness for emerging AMR threats within New Zealand’s available resources [19,110, 111].

### Local epidemiology and lack of patient-to-patient transmission

WGS revealed a heterogeneous population structure from the clinical isolates, with 75 distinct STs identified among 121 high-quality genomes. Most STs were represented by a single isolate, while a small number of STs recurred intermittently over the study period. Apparent phylogenetic ‘clusters’ (Figs3  4) may reflect sampling of clones that are circulating more broadly within the local community rather than transmission within the hospital setting: within repeated STs, pairwise SNV distances exceeded thresholds typically associated with *K. pneumoniae* transmission (≤20 to 25 SNVs, relaxed to <50 SNVs for Oxford Nanopore R9.4 flow cell data [45,107, 108]) and far greater than would be expected from a single transmission chain [12,13, 112]. Windows of 0× read depth, observed in most pairwise comparisons, indicate regions of poor homology between genomes and likely reflect underlying sequence divergence rather than technical dropout. Accordingly, even though pairwise distances fall within the relaxed <50 SNV threshold proposed for Oxford Nanopore-only data [45], these missing regions suggest that the compared strains may, in fact, be more genetically distinct than their SNV counts imply. Only two STs displayed consistently complete chromosomal coverage: ST253, consistent with within-patient persistence (Fig. S3), and ST557, suggesting a possible community or environmental source (Fig. S4). Although genomes collected within the same hospital and year sometimes differed by several hundred SNVs, these long branch lengths likely reflect the heterogeneous population structure and incomplete sampling. These findings align with genomic surveillance studies from Australia [13] and Norway [113], which also reported multi-lineage hospital populations with low transmission burden. In these settin*g*s, many *K. pneumoniae* infections reflect overgrowth of diverse gut colonizing strains acquired outside of the hospital rather than persistence of endemic hospital lineages. This distinction is crucial: it links prevention priorities to phase-based responses grounded in sustainability and risk mitigation; progressing from elimination to aggressive (‘stamp-it-out’) containment and, when warranted, to long-term management, supported by integrated strategies addressing screening policies, colonization, community reservoirs and antimicrobial stewardship.

### A stable chromosomal core beneath a mobile ‘resistance-ready’ plasmidome

The chromosomal resistome of local clinical/screened *K. pneumoniae* was dominated by intrinsic loci, consistent with its characteristic baseline resistance to ampicillin and low-level tolerance to fosfomycin and quinolones [12,14]. The absence of chromosomal *amp*C, as expected for *K. pneumoniae*, underscores its dependence on plasmid acquisition for extended-spectrum cephalosporin resistance [14,114]. Among the 118 complete genomes, extended-spectrum cephalosporin resistance appeared to be driven primarily by CTX-M-type ESBL enzymes (Tables S6, S11 and S12), consistent with global patterns where CTX-M-15 predominates and typically rides on F-type or mosaic plasmids with co-resistance cargo [14,47, 112]. Notably, only a single carbapenemase gene was detected, consistent with the high rate of phenotypic susceptibility to carbapenems. The overall picture is one of limited endemic ESBL circulation relative to high-burden settings, with signals of ongoing plasmid-mediated exchange rather than clonal expansion.

In contrast to the relatively stable chromosomal background, plasmid diversity was greater. F-type and hybrid mosaic plasmids predominated, followed by smaller Col replicons. The predominance of singleton plasmid subcommunities, together with the absence of conserved multidrug-resistance architectures across STs, suggests episodic plasmid acquisition rather than stable in-hospital plasmid lineages. These plasmid distributions mirror global patterns, where F-type backbones act as major carriers of ESBLs and carbapenemase genes, while Col plasmids often serve as accessory vectors for resistance and virulence [12,14,32, 113]. Reconstruction of representative F-type plasmids revealed near-identity to international ESBL- and carbapenemase-associated backbones but with most resistance loci absent, retaining only *tet*(A), *qnr*B1 or *dfr*A14 (see the ‘Resistance-sparse F-type plasmids predominate despite similarity to global multidrug resistance backbones’ section). These findings show that although carbapenem resistance remains rare [61], high-risk plasmid scaffolds are already circulating locally in a largely susceptible form, providing a genomic platform for possible future acquisition and dissemination of clinically significant AMR genes [12,14,109]. Comparison with international references indicates that these New Zealand plasmids differ primarily by the presence or absence of IS*26*-linked AMR cassettes, confirming that similar backbones elsewhere have already captured and mobilized resistance loci; evidence that these local plasmids retain the same genetic capacity for AMR acquisition.

Structural comparisons revealed near-identical arrangements of IS*26*-Tn*3* arrangements in two ST15 genomes (albeit sourced from the same patient), supporting *in situ* chromosomal expansion of a composite resistance region carrying quinolone (*qnr*B1), beta-lactam (*bla*_SHV-12_; *bla*_OXA-1_), phenicols (*cat*B3) and aminoglycoside [*aac*(6′)-*Ib-cr*] resistance determinants (Fig. S2). These arrays may enable copy-number variation of embedded resistance determinants. While phenotypic effects were not evaluated here, expansion of such IS*26*-linked modules has been associated elsewhere with increased beta-lactamase dosage and elevated MICs, suggesting a potential rapid-tuning mechanism for beta-lactam resistance [116,118]. This copy-number plasticity offers a route to within-host adaptation even over short timescales and (because the module is chromosomal) decouples resistance stability from plasmid maintenance. Clinically, single-colony workflows may miss such copy-number variants capable of traversing susceptible, intermediate and non-susceptible breakpoints. As such, integrating genome structure (including IS-mediated copy-number variation) with phenotypes is important. As Vitek^®^ 2 reports discrete concentration steps (‘MIC equivalents’) rather than reference broth-microdilution MICs, our data demonstrate association rather than causation. Nonetheless, the concordant structural and phenotypic shifts implicate IS*26*-driven amplification as a modulator of beta-lactam susceptibility *in vivo*.

More broadly, these IS-driven events demonstrate that chromosomal capture of plasmid-derived resistance modules can occur in *Enterobacterales*, creating stable multidrug-resistance islands when conditions favour retention [12,14, 32]. However, such events appear to remain relatively uncommon, and current evidence does not indicate a wider shift towards increasing fixation. In a separate investigation of *Escherichia coli* ST131, IS*1*-mediated integration of *bla*_OXA-48_ into a Col156 plasmid facilitated subsequent chromosomal insertion [115], illustrating a similar dynamic.

Together, these findings depict a resistance landscape defined by a largely intrinsic chromosomal background, overlaid by the circulation of multi-replicon plasmids with broad adaptive potential. While overt resistance remains uncommon in our setting, plasmid frameworks associated with AMR are present (in clinical samples at least), highlighting the potential value of investment in AMR surveillance even in low-burden settings [12,14,22, 113].

### Virulence ecology and the absence of convergence

The virulence profile mirrored the low-resistance landscape: accessory content was limited; markers of virulence plasmids (*iuc*/*iro* with *rmp*A*/rmp*A2; Kleborate virulence score ≥3), which are associated with hypervirulent phenotypes, were rare; and none co-occurred with acquired AMR (Table S5). Convergence arises when a strain or plasmid acquires genes for resistance alongside key virulence loci [32], often through mobile genetic elements [119,122]. Globally, the emergence of these ‘convergent’ clones drives invasive, treatment-refractory infections and blurs the long-standing boundary between multidrug-resistant and hypervirulent populations [12,14,30, 32, 42, 47, 119, 120, 122]. No evidence of convergence was found in clinical/screening isolates from this study. The local population appears genetically diverse, largely drug-susceptible and comprised of a range of lineages. This is reassuring yet fragile; plasmid frameworks capable of capturing virulence or resistance genes are already circulating locally. Decentralized, real-time sequencing may provide the means to detect the incursion of a dual-threat clone before it becomes entrenched.

### Study limitations and strengths

This pilot presents a proof-of-concept for decentralized genomic surveillance of *K. pneumoniae* in New Zealand, conducted at a tertiary hospital serving ~500,000 people. While valuable, the scope was necessarily constrained: sampling was limited to a single institution (i.e. Wellington Regional Hospital) and a finite 15-month window beginning in January 2022, meaning the study provides a snapshot of activity but not the broader temporal or national context. Only clinical isolates were sequenced, as the immediate clinical objective was species identification and MLST to support proactive surveillance [22,56,58, 63]. Although several isolates shared a cgST, pairwise SNV distances and genome-wide variation patterns indicated independent introductions rather than recent transmission; consequently, no genomic clusters consistent with hospital spread were detected, and no infection-prevention investigations were triggered that would have included carriage or environmental sampling [22]. A complementary One Health component, such as environmental or colonization sampling, would have strengthened the study, but was beyond the initial scope.

Additionally, classification of cases as community- or hospital-onset was based on a 72-h threshold relative to admission. While this approach increases specificity for healthcare-associated acquisition, in the absence of systematic admission screening, some cases classified as hospital-onset may represent community-acquired organisms detected later during admission.

The dataset was modest (121 genomes retained for SNV-based analysis after quality filtering). A total of 36 additional isolates did not meet the coverage/depth thresholds required for high-resolution SNV-based phylogenetic analysis and were, therefore, excluded from this component of the study (Table S3). Importantly, these datasets were not sequencing failures; some cases still yielded information for lower-resolution analyses (e.g. MLST), which was the primary clinical output at the time of sequencing [63]. As such, re-sequencing was not routinely performed, as this would have increased cost and turnaround time without improving the intended use of the data. Nonetheless, the exclusion of these genomes from SNV-based analyses may bias estimates of diversity, transmission and resistance.

Importantly, the study relied entirely on Oxford Nanopore sequencing, which offers both advantages and caveats. ‘Any-length’ reads enabled near-complete genome assemblies and resolution of plasmids, integrative elements and repetitive regions that are often fragmented in short-read data. This enabled confident reconstruction of plasmid backbones and chromosomal AMR or virulence insertions, delivering structural resolution that is rarely achievable through short-read surveillance alone [45,68]. Basecalling used Dorado neural-network models; consensus assemblies were generated with Autocycler from the basecalled reads and polished from read mappings, while small-variant detection used Clair3 neural-network models on the mapped reads. However, even with super-accuracy basecalling and assembly polishing, residual base-level errors can persist in R9.4 data, particularly in homopolymer regions. This is important for loci such as capsule and virulence genes, where apparent frameshifts or premature stop codons may reflect sequencing or assembly artefacts rather than true biological disruption. The high frequency of predicted capsule-null K loci observed here is consistent with this limitation [45]. Accordingly, Nanopore-only R9.4 assemblies were highly informative for lineage assignment, plasmid reconstruction and transmission analysis, but were less reliable for interpreting putative gene truncation or loss-of-function in homopolymer-rich loci. Despite this, previous work has shown that such data can still support accurate genomic clustering analyses to indicate or rule out putative transmission events [45,59, 68].

Finally, the additional onsite workload yielded no immediate impact on infection control in this low-resistance setting, and bioinformatics support remained centralized. Collectively, these caveats emphasize the need for sustained, multicentre, One Health-integrated surveillance to complement the local baseline established here. New Zealand’s distinctive context (geographic isolation and persistently low carbapenemase prevalence [61,62]) makes this study a valuable ‘natural experiment’, while also limiting direct extrapolation to high-resistance, high-burden settings.

Despite these limitations, this study has several strengths. It represents the first prospective, decentralized, nanopore-driven genomic monitoring of *K. pneumoniae* in New Zealand, integrating real-time sequencing within a diagnostic laboratory [56]. By sequencing bacterial genomes onsite, the hospital was able to investigate a broader range of *K. pneumoniae* infections (including drug-susceptible strains that might not be referred under national surveillance standards) instead of depending on centralized systems that focus on nationally important threats like carbapenemase producers. This offers early-warning capabilities and guarantees that locally significant issues can be prioritized for investigation at a local level [22]. The prospective design enabled proactive surveillance across 15 months, in contrast to point prevalence surveys, and produced 118 complete *K. pneumoniae* genomes. These high-quality assemblies facilitated strong characterization of chromosomal, plasmid, resistance and virulence loci, while capturing consecutive clinical cases in all age groups to create an impartial baseline of local diversity. The combination of onsite sequencing with centralized analytic support demonstrated the practicality of a decentralized model that could be expanded across hospitals both nationally and internationally. Importantly, the data collected in this low-resistance setting provides a genomic baseline for future surveillance of high-risk clone incursions. By depositing all sequence data and genome assemblies in the NCBI (BioProject PRJNA1332379), this work also contributes a durable resource for international comparison and global surveillance. In doing so, this pilot demonstrates how decentralized, hospital-embedded genomics can set a new standard for proactive pathogen surveillance, and we welcome others to make use of these publicly available resources. This will help shift infection surveillance from reactive containment towards proactive and coordinated prevention of emerging AMR threats.

### Conclusions

This study provides the first prospective genomic snapshot of clinical *K. pneumoniae* in a New Zealand hospital, revealing a highly diverse, largely drug-susceptible population with no evidence of sustained patient–patient transmission detected during the study period. Embedding nanopore sequencing directly within a diagnostic laboratory and linking these data to national analytic expertise demonstrates the feasibility of decentralized, hospital-embedded genomics in a low-burden setting. This pilot offers a practical foundation for a scalable, networked model of real-time pathogen surveillance that complements existing national reference systems. Sustained investment in such distributed genomic capability will strengthen New Zealand’s preparedness for emerging antimicrobial-resistant and healthcare-associated threats, while contributing to global efforts to move infection control from reactive response to proactive prevention.

## Supplementary material

10.1099/mgen.0.001700 Uncited Fig. S1.

10.1099/mgen.0.001700 Uncited Table S1.

## Abbreviations

AMR: antimicrobial resistance
CG: clonal group
cgST: core-genome sequence type
DHB: District Health Board
ESBL: extended-spectrum beta-lactamase
GLASS: Global Antimicrobial Resistance and Use Surveillance System
ICE: integrative conjugative element
Indel: insertion or deletion
IPC: infection prevention control
IQR: interquartile range
KpSC: *Klebsiella pneumoniae* species complex
LIN: Life Identification Number
MIC: minimum inhibitory concentration
MLST: multilocus sequence typing
NCBI: National Center for Biotechnology Information
PGAP: Prokaryotic Genome Annotation Pipeline
PHF Science: Public Health and Forensic Science
RefSeq: Reference Sequence
S/I/R: susceptible/intermediate/resistant
SNV: single-nucleotide variant
STs: sequence types
WGS: whole-genome sequencing

## References

[1] Murray CJL, Ikuta KS, Sharara F, Swetschinski L, Robles Aguilar G (2022). Global burden of bacterial antimicrobial resistance in 2019: a systematic analysis. The Lancet.

[2] Manesh A, Shankar C, George MM, Jasrotia DS, Lal B (2023). Clinical and genomic evolution of carbapenem-resistant *Klebsiella pneumoniae* bloodstream infections over two time periods at a tertiary care hospital in South India: a prospective cohort study. Infect Dis Ther.

[3] O’Neill J (2016). Tackling drug-resistant infections globally: final report and recommendations. https://amr-review.org/.

[4] Jonas OB, Irwin A, Le Gall FG, Marquez PV (2017). Drug-resistant infections: a threat to our economic future. http://documents.worldbank.org/curated/en/323311493396993758.

[5] Tacconelli E, Carrara E, Savoldi A, Harbarth S, Mendelson M (2018). Discovery, research, and development of new antibiotics: the WHO priority list of antibiotic-resistant bacteria and tuberculosis. Lancet Infect Dis.

[6] Wyres KL, Holt KE (2018). Klebsiella pneumoniae as a key trafficker of drug resistance genes from environmental to clinically important bacteria. Curr Opin Microbiol.

[7] World Health Organization (2022). Global antimicrobial resistance and use surveillance system (GLASS) report. https://www.who.int/publications/i/item/9789240108127.

[8] Friedlaender C (1882). Ueber die Schizomyceten bei der acuten fibrösen Pneumonie. Archiv f pathol Anat.

[9] Pendleton JN, Gorman SP, Gilmore BF (2013). Clinical relevance of the ESKAPE pathogens. Expert Rev Anti Infect Ther.

[10] Podschun R, Ullmann U (1998). Klebsiella spp. as nosocomial pathogens: epidemiology, taxonomy, typing methods, and pathogenicity factors. Clin Microbiol Rev.

[11] Magill SS, Edwards JR, Bamberg W, Beldavs ZG, Dumyati G (2014). Multistate point-prevalence survey of health care–associated infections. N Engl J Med.

[12] Lam MMC, Wick RR, Watts SC, Cerdeira LT, Wyres KL (2021). A genomic surveillance framework and genotyping tool for *Klebsiella pneumoniae* and its related species complex. Nat Commun.

[13] Gorrie CL, Mirčeta M, Wick RR, Judd LM, Lam MMC (2022). Genomic dissection of *Klebsiella pneumoniae* infections in hospital patients reveals insights into an opportunistic pathogen. Nat Commun.

[14] Wyres KL, Lam MMC, Holt KE (2020). Population genomics of *Klebsiella pneumoniae*. Nat Rev Microbiol.

[15] Gorrie CL, Mirceta M, Wick RR, Edwards DJ, Thomson NR (2017). Gastrointestinal carriage is a major reservoir of *Klebsiella pneumoniae* infection in intensive care patients. Clin Infect Dis.

[16] Martin RM, Cao J, Brisse S, Passet V, Wu W (2016). Molecular epidemiology of colonizing and infecting isolates of *Klebsiella pneumoniae*. *mSphere*.

[17] Shimasaki T, Seekatz A, Bassis C, Rhee Y, Yelin RD (2019). Increased relative abundance of *Klebsiella pneumoniae* carbapenemase-producing *Klebsiella pneumoniae* within the gut microbiota Is associated with risk of bloodstream infection in long-term acute care hospital patients. Clin Infect Dis.

[18] World Health Organization (2025). Global antibiotic resistance surveillance report 2025. https://www.who.int/publications/i/item/9789240116337/.

[19] Ministry of Health (2025). Public Health Surveillance Strategy 2025-2030. https://www.health.govt.nz/publications/public-health-surveillance-strategy-2025-2030.

[20] Rodríguez-Medina N, Barrios-Camacho H, Duran-Bedolla J, Garza-Ramos U (2019). *Klebsiella variicola*: an emerging pathogen in humans. Emerg Microbes Infect.

[21] de Campos TA, de Almeida FM, de Almeida APC, Nakamura-Silva R, Oliveira-Silva M (2021). Multidrug-Resistant (MDR) *Klebsiella variicola* strains isolated in a Brazilian hospital belong to new clones. Front Microbiol.

[22] White RT, Balm M, Burton M, Hutton S, Jeram J (2025). The rapid detection of a neonatal unit outbreak of a wild-type *Klebsiella variicola* using decentralized Oxford Nanopore sequencing. *Antimicrob Resist Infect Control*.

[23] Rodrigues C, Passet V, Rakotondrasoa A, Brisse S (2018). Identification of *Klebsiella pneumoniae, Klebsiella quasipneumoniae, Klebsiella variicola* and Related Phylogroups by MALDI-TOF Mass Spectrometry. Front Microbiol.

[24] Rodrigues C, Passet V, Rakotondrasoa A, Diallo TA, Criscuolo A (2019). *Klebsiella variicola* subsp. *tropicalensis* subsp nov and *klebsiella variicola* subsp *variicola* subsp nov. Res Microbiol.

[25] Maatallah M, Vading M, Kabir MH, Bakhrouf A, Kalin M (2014). *Klebsiella variicola* is a frequent cause of bloodstream infection in the stockholm area, and associated with higher mortality compared to *K. pneumoniae*. PLoS One.

[26] Brisse S, Verhoef J (2001). Phylogenetic diversity of *Klebsiella pneumoniae* and *Klebsiella oxytoca* clinical isolates revealed by randomly amplified polymorphic DNA, gyrA and parC genes sequencing and automated ribotyping. Int J Syst Evol Microbiol.

[27] Rosenblueth M, Martínez L, Silva J, Martínez-Romero E (2004). *Klebsiella variicola*, a novel species with clinical and plant-associated isolates. Syst Appl Microbiol.

[28] Brisse S, Passet V, Grimont PAD (2014). Description of *Klebsiella quasipneumoniae* sp. nov., isolated from human infections, with two subspecies, *Klebsiella quasipneumoniae* subsp. *quasipneumoniae* subsp. nov. and *Klebsiella quasipneumoniae* subsp. *similipneumoniae* subsp. nov., and demonstration that Klebsiella singaporensis is a junior heterotypic synonym of *Klebsiella variicola*. Int J Syst Evol Microbiol.

[29] Bialek-Davenet S, Criscuolo A, Ailloud F, Passet V, Jones L (2014). Genomic definition of hypervirulent and multidrug-resistant *Klebsiella pneumoniae* clonal groups. Emerg Infect Dis.

[30] Holt KE, Wertheim H, Zadoks RN, Baker S, Whitehouse CA (2015). Genomic analysis of diversity, population structure, virulence, and antimicrobial resistance in *Klebsiella pneumoniae*, an urgent threat to public health. Proc Natl Acad Sci U S A.

[31] Long SW, Linson SE, Ojeda Saavedra M, Cantu C, Davis JJ (2017). Whole-genome sequencing of a human clinical isolate of the novel species *Klebsiella quasivariicola* sp. nov. Genome Announc.

[32] Wyres KL, Wick RR, Judd LM, Froumine R, Tokolyi A (2019). Distinct evolutionary dynamics of horizontal gene transfer in drug resistant and virulent clones of *Klebsiella pneumoniae*. PLoS Genet.

[33] Orskov I, Fife-asbury MA (1977). New Klebsiella capsular antigen, K82, and the deletion of five of those previously assigned. Int J Syst Bacteriol.

[34] Trautmann M, Ruhnke M, Rukavina T, Held TK, Cross AS (1997). O-antigen seroepidemiology of Klebsiella clinical isolates and implications for immunoprophylaxis of Klebsiella infections. Clin Diagn Lab Immunol.

[35] Elhani D, Bakir L, Aouni M, Passet V, Arlet G (2010). Molecular epidemiology of extended-spectrum beta-lactamase-producing *Klebsiella pneumoniae* strains in a university hospital in Tunis, Tunisia, 1999-2005. Clin Microbiol Infect.

[36] Chen L, Mathema B, Chavda KD, DeLeo FR, Bonomo RA (2014). Carbapenemase-producing *Klebsiella pneumoniae*: molecular and genetic decoding. Trends Microbiol.

[37] Diancourt L, Passet V, Verhoef J, Grimont PAD, Brisse S (2005). Multilocus sequence typing of *Klebsiella pneumoniae* nosocomial isolates. J Clin Microbiol.

[38] Brisse S, Fevre C, Passet V, Issenhuth-Jeanjean S, Tournebize R (2009). Virulent clones of Klebsiella pneumoniae: identification and evolutionary scenario based on genomic and phenotypic characterization. PLoS One.

[39] Palmieri M, Wyres KL, Mirande C, Qiang Z, Liyan Y (2019). Genomic evolution and local epidemiology of *Klebsiella pneumoniae* from a major hospital in Beijing, China, over a 15 year period: dissemination of known and novel high-risk clones. Microb Genom.

[40] Wyres KL, Holt KE (2016). *Klebsiella pneumoniae* population genomics and antimicrobial-resistant clones. Trends Microbiol.

[41] Paczosa MK, Mecsas J (2016). *Klebsiella pneumoniae*: going on the offense with a strong defense. Microbiol Mol Biol Rev.

[42] Lam MMC, Wick RR, Wyres KL, Gorrie CL, Judd LM (2018). Genetic diversity, mobilisation and spread of the yersiniabactin-encoding mobile element ICEKp in *Klebsiella pneumoniae* populations. Microb Genom.

[43] Russo TA, Marr CM (2019). Hypervirulent *Klebsiella pneumoniae*. Clin Microbiol Rev.

[44] Siu LK, Yeh KM, Lin JC, Fung CP, Chang FY (2012). *Klebsiella pneumoniae* liver abscess: a new invasive syndrome. Lancet Infect Dis.

[45] Foster-Nyarko E, Cottingham H, Wick RR, Judd LM, Lam MMC (2023). Nanopore-only assemblies for genomic surveillance of the global priority drug-resistant pathogen, *Klebsiella pneumoniae*. Microb Genom.

[46] World Health Organization (2016). Global action plan on antimicrobial resistance. https://www.who.int/publications/i/item/9789241509763.

[47] Wyres KL, Nguyen TNT, Lam MMC, Judd LM, van Vinh Chau N (2020). Genomic surveillance for hypervirulence and multi-drug resistance in invasive *Klebsiella pneumoniae* from South and Southeast Asia. Genome Med.

[48] Cassini A, Högberg LD, Plachouras D, Quattrocchi A, Hoxha A (2019). Attributable deaths and disability-adjusted life-years caused by infections with antibiotic-resistant bacteria in the EU and the European Economic Area in 2015: a population-level modelling analysis. Lancet Infect Dis.

[49] Nagaraj G, Shamanna V, Govindan V, Rose S, Sravani D (2021). High-resolution genomic profiling of carbapenem-resistant *Klebsiella pneumoniae* isolates: a multicentric retrospective indian study. Clin Infect Dis.

[50] Saavedra SY, Bernal JF, Montilla-Escudero E, Arévalo SA, Prada DA (2021). Complexity of genomic epidemiology of carbapenem-resistant *Klebsiella pneumoniae* isolates in Colombia Urges the reinforcement of whole genome sequencing-based surveillance programs. Clin Infect Dis.

[51] Aanensen DM, Carlos CC, Donado-Godoy P, Okeke IN, Ravikumar KL (2021). Implementing whole-genome sequencing for ongoing surveillance of antimicrobial resistance: exemplifying insights Into *Klebsiella pneumoniae*. Clin Infect Dis.

[52] Heffernan H, Woodhouse R, Draper J, Ren X (2016). survey of extended-spectrum β-lactamaseproducing Enterobacteriaceae. https://www.phfscience.nz/digital-library/2016-survey-of-extended-spectrum-%CE%B2-lactamase-esbl-producing-enterobacteriaceae/.

[53] Tiong A, Woodhouse R, White R, Winter D, Dyet K (2019). survey of extended-spectrum β-lactamase producing Enterobacterales in New Zealand. https://www.phfscience.nz/digital-library/2019-survey-of-extended-spectrum-%CE%B2-lactamase-esbl-producing-enterobacterales-in-new-zealand/.

[54] Carr S, Bakker S, Eustace A, Elvy J, Gilkison C (2021). survey of Staphylococcus aureus bacteraemia in New Zealand. https://www.phfscience.nz/digital-library/2021-survey-of-staphylococcus-aureus-bacteraemia-in-new-zealand/.

[55] Stewart J, Judd LM, Jenney A, Holt KE, Wyres KL (2022). Epidemiology and genomic analysis of Klebsiella oxytoca from a single hospital network in Australia. BMC Infect Dis.

[56] Manning S, Bloomfield M, Hutton S, Burton M, Velasco C (2025). Implementing nanopore sequencing in a clinical laboratory: a social systems case New Zealand. Journal of Medical Laboratory Science.

[57] White RT, Bakker S, Burton M, Castro ML, Couldrey C (2024). Rapid identification and subsequent contextualization of an outbreak of methicillin-resistant *Staphylococcus aureus* in a neonatal intensive care unit using nanopore sequencing. Microb Genom.

[58] Bloomfield M, Hutton S, Burton M, Tarring C, Velasco C (2024). Early identification of a ward-based outbreak of *Clostridioides difficile* using prospective multilocus sequence type-based Oxford Nanopore genomic surveillance. Infect Control Hosp Epidemiol.

[59] Bloomfield M, Bakker S, Burton M, Castro ML, Dyet K (2025). Resolving a neonatal intensive care unit outbreak of meticillin-resistant *Staphylococcus aureus* to the single-nucleotide variant level using Oxford Nanopore simplex reads and HERRO error correction. Journal of Hospital Infection.

[60] Hall MB, Wick RR, Judd LM, Nguyen AN, Steinig EJ (2024). Benchmarking reveals superiority of deep learning variant callers on bacterial nanopore sequence data. Elife.

[61] Institute of Environmental Science and Research (2025). Carbapenemase-producing Enterobacterales (CPE) in New Zealand. https://www.phfscience.nz/digital-library/2022-enterobacterales-with-acquired-carbapenemases/.

[62] Thornley CN, Kelly M, Bloomfield M, Mangalasseril L, Nesdale A (2025). Community outbreak of OXA-48-producing *Escherichia coli* linked to food premises, New Zealand, 2018-2022. Emerg Infect Dis.

[63] Bloomfield M, Hutton S, Velasco C, Burton M, Benton M (2024). Oxford nanopore next generation sequencing in a front-line clinical microbiology laboratory without on-site bioinformaticians. Pathology.

[64] European Committee on Antimicrobial Susceptibility Testing (2023). Breakpoint tables for interpretation of MICs and zone diameters. Version 13.1.

[65] De Coster W, D’Hert S, Schultz DT, Cruts M, Van Broeckhoven C (2018). NanoPack: visualizing and processing long-read sequencing data. Bioinformatics.

[66] Wood DE, Salzberg SL (2014). Kraken: ultrafast metagenomic sequence classification using exact alignments. Genome Biol.

[67] Sayers EW, Barrett T, Benson DA, Bryant SH, Canese K (2009). Database resources of the National Center for Biotechnology Information. Nucleic Acids Res.

[68] Wick RR, Howden BP, Stinear TP (2025). Autocycler: long-read consensus assembly for bacterial genomes. Bioinformatics.

[69] Koren S, Walenz BP, Berlin K, Miller JR, Bergman NH (2017). Canu: scalable and accurate long-read assembly via adaptive *k*-mer weighting and repeat separation. Genome Res.

[70] Kolmogorov M, Yuan J, Lin Y, Pevzner PA (2019). Assembly of long, error-prone reads using repeat graphs. Nat Biotechnol.

[71] Li H (2016). Minimap and miniasm: fast mapping and de novo assembly for noisy long sequences. Bioinformatics.

[72] Hu J, Wang Z, Sun Z, Hu B, Ayoola AO (2024). NextDenovo: an efficient error correction and accurate assembly tool for noisy long reads. Genome Biol.

[73] Vaser R, Sikic M (2021). Time- and memory-efficient genome assembly with raven. Nature Computational Science.

[74] Li H (2018). Minimap2: pairwise alignment for nucleotide sequences. Bioinformatics.

[75] Li H (2021). New strategies to improve minimap2 alignment accuracy. Bioinformatics.

[76] Vaser R, Sović I, Nagarajan N, Šikić M (2017). Fast and accurate de novo genome assembly from long uncorrected reads. Genome Res.

[77] Bouras G, Grigson SR, Papudeshi B, Mallawaarachchi V, Roach MJ (2024). Dnaapler: a tool to reorient circular microbial genomes. JOSS.

[78] Tatusova T, DiCuccio M, Badretdin A, Chetvernin V, Nawrocki EP (2016). NCBI prokaryotic genome annotation pipeline. Nucleic Acids Res.

[79] Haft DH, DiCuccio M, Badretdin A, Brover V, Chetvernin V (2018). RefSeq: an update on prokaryotic genome annotation and curation. Nucleic Acids Res.

[80] Li W, O’Neill KR, Haft DH, DiCuccio M, Chetvernin V (2021). RefSeq: expanding the Prokaryotic Genome Annotation Pipeline reach with protein family model curation. Nucleic Acids Res.

[81] Gladstone RA, Lo SW, Goater R, Yeats C, Taylor B (2020). Visualizing variation within Global Pneumococcal Sequence Clusters (GPSCs) and country population snapshots to contextualize pneumococcal isolates. Microb Genom.

[82] Jolley KA, Bray JE, Maiden MCJ (2018). Open-access bacterial population genomics: BIGSdb software, the PubMLST.org website and their applications. Wellcome Open Res.

[83] Wyres KL, Wick RR, Gorrie C, Jenney A, Follador R (2016). Identification of Klebsiella capsule synthesis loci from whole genome data. Microb Genom.

[84] Lam MMC, Wick RR, Judd LM, Holt KE, Wyres KL (2022). Kaptive 2.0: updated capsule and lipopolysaccharide locus typing for the *Klebsiella pneumoniae* species complex. Microb Genom.

[85] Stanton TD, Hetland MAK, Löhr IH, Holt KE, Wyres KL (2025). Fast and accurate in silico antigen typing with Kaptive 3. Microb Genom.

[86] Feldgarden M, Brover V, Gonzalez-Escalona N, Frye JG, Haendiges J (2021). AMRFinderPlus and the Reference Gene Catalog facilitate examination of the genomic links among antimicrobial resistance, stress response, and virulence. Sci Rep.

[87] Carattoli A, Zankari E, García-Fernández A, Voldby Larsen M, Lund O (2014). In silico detection and typing of plasmids using PlasmidFinder and plasmid multilocus sequence typing. Antimicrob Agents Chemother.

[88] Carattoli A, Hasman H (2020). PlasmidFinder and In Silico pMLST: Identification and Typing of Plasmid Replicons in Whole-Genome Sequencing (WGS). *Methods Mol Biol*.

[89] Robertson J, Nash JHE (2018). MOB-suite: software tools for clustering, reconstruction and typing of plasmids from draft assemblies. Microb Genom.

[90] Robertson J, Bessonov K, Schonfeld J, Nash JHE (2020). Universal whole-sequence-based plasmid typing and its utility to prediction of host range and epidemiological surveillance. Microb Genom.

[91] Ondov BD, Treangen TJ, Melsted P, Mallonee AB, Bergman NH (2016). Mash: fast genome and metagenome distance estimation using minhash. Genome Biol.

[92] Tian L, Huang C, Mazloom R, Heath LS, Vinatzer BA (2020). LINbase: a web server for genome-based identification of prokaryotes as members of crowdsourced taxa. Nucleic Acids Res.

[93] Hennart M, Guglielmini J, Bridel S, Maiden MCJ, Jolley KA (2022). A dual barcoding approach to bacterial strain nomenclature: genomic taxonomy of klebsiella pneumoniae strains. Mol Biol Evol.

[94] Raabe NJ, Griffith MP, Srinivasa VR, Waggle KD, Sundermann AJ (2025). ACCIO: an assembly-based tool enabling plasmid detection. *medRxiv*.

[95] Frolova D, Lima L, Roberts LW, Bohnenkamper L, Wittler R (2024). Applying rearrangement distances to enable plasmid epidemiology with pling. Microb Genom.

[96] Li H, Handsaker B, Wysoker A, Fennell T, Ruan J (2009). The Sequence Alignment/Map format and SAMtools. Bioinformatics.

[97] Zheng Z, Li S, Su J, Leung AW-S, Lam T-W (2022). Symphonizing pileup and full-alignment for deep learning-based long-read variant calling. *Nat Comput Sci*.

[98] Danecek P, Bonfield JK, Liddle J, Marshall J, Ohan V (2021). Twelve years of SAMtools and BCFtools. Gigascience.

[99] R Core Team (2021). R: A Language and Environment for Statistical Computing.

[100] Wickham H (2016). Ggplot2: Elegant Graphics for Data Analysis New York, United States of America.

[101] Sarovich DS, Price EP (2014). SPANDx: a genomics pipeline for comparative analysis of large haploid whole genome re-sequencing datasets. BMC Res Notes.

[102] Croucher NJ, Page AJ, Connor TR, Delaney AJ, Keane JA (2015). Rapid phylogenetic analysis of large samples of recombinant bacterial whole genome sequences using Gubbins. Nucleic Acids Res.

[103] Quinlan AR, Hall IM (2010). BEDTools: a flexible suite of utilities for comparing genomic features. Bioinformatics.

[104] Wilgenbusch JC, Swofford D (2003). Inferring evolutionary trees with PAUP*. Curr Protoc Bioinformatics.

[105] Lam MMC, Wyres KL, Judd LM, Wick RR, Jenney A (2018). Tracking key virulence loci encoding aerobactin and salmochelin siderophore synthesis in *Klebsiella pneumoniae*. Genome Med.

[106] Lam MMC, Salisbury SM, Treat LP, Wick RR, Judd LM (2025). Genomic and functional analysis of rmp locus variants in *Klebsiella pneumoniae*. Genome Med.

[107] Sherry NL, Lane CR, Kwong JC, Schultz M, Sait M (2019). Genomics for molecular epidemiology and detecting transmission of carbapenemase-producing *Enterobacterales* in Victoria, Australia, 2012 to 2016. J Clin Microbiol.

[108] David S, Reuter S, Harris SR, Glasner C, Feltwell T (2019). Epidemic of carbapenem-resistant *Klebsiella pneumoniae* in Europe is driven by nosocomial spread. Nat Microbiol.

[109] Sundermann AJ, Kumar P, Griffith MP, Waggle KD, Rangachar Srinivasa V (2026). Real-time genomic surveillance for enhanced healthcare outbreak detection and control: clinical and economic impact. Clin Infect Dis.

[110] Gerrard J (2021). Kotahitanga: Uniting Aotearoa against infectious disease and antimicrobial resistance. https://www.pmcsa.ac.nz/topics/antimicrobial-resistance-and-infectious-disease/.

[111] Ministry of Health (2025). Government response to the report from the Prime Minister’s Chief Science Advisor: Kotahitanga: Uniting Aotearoa against infectious disease and antimicrobial resistance. https://www.health.govt.nz/publications/government-response-to-the-report-from-the-prime-ministers-chief-science-advisor.

[112] Hawkey J, Wyres KL, Judd LM, Harshegyi T, Blakeway L (2022). ESBL plasmids in *Klebsiella pneumoniae*: diversity, transmission and contribution to infection burden in the hospital setting. Genome Med.

[113] Fostervold A, Hetland MAK, Bakksjø R, Bernhoff E, Holt KE (2022). A nationwide genomic study of clinical *Klebsiella pneumoniae* in Norway 2001-15: introduction and spread of ESBLs facilitated by clonal groups CG15 and CG307. J Antimicrob Chemother.

[114] Gorrie CL, Mirceta M, Wick RR, Judd LM, Wyres KL (2018). Antimicrobial-resistant *Klebsiella pneumoniae* carriage and infection in specialized geriatric care wards linked to acquisition in the referring hospital. Clin Infect Dis.

[115] White RT, Thornley CN, Bloomfield M, Dyet K, Elvy J (2026). Integration of bla_OXA-48_ into a Col156 plasmid drove a carbapenem-resistant Escherichia coli ST131 outbreak in New Zealand: global genomic evidence for the gene’s multilayered dissemination. Drug Resist Updat.

[116] Hubbard ATM, Mason J, Roberts P, Parry CM, Corless C (2020). Piperacillin/tazobactam resistance in a clinical isolate of *Escherichia coli* due to IS26-mediated amplification of bla_TEM-1B_. Nat Commun.

[117] Forde BM, Henderson A, Playford EG, Looke D, Henderson BC (2021). Fatal respiratory diphtheria caused by ß-Lactam-resistant *Corynebacterium diphtheriae*. Clin Infect Dis.

[118] White RT, Ashcroft MM, Bauer MJ, Bell J, Butkiewicz D (2024). The complete genome sequence of five pre-2013 *Escherichia coli* sequence type (ST)1193 strains reveals insights into an emerging pathogen. Access Microbiology.

[119] Giske CG, Monnet DL, Cars O, Carmeli Y (2008). Clinical and economic impact of common multidrug-resistant gram-negative Bacilli. Antimicrob Agents Chemother.

[120] Jung Y, Lee MJ, Sin H-Y, Kim N-H, Hwang J-H (2012). Differences in characteristics between healthcare-associated and community-acquired infection in community-onset Klebsiella pneumoniae bloodstream infection in Korea. BMC Infect Dis.

[121] Navon-Venezia S, Kondratyeva K, Carattoli A (2017). *Klebsiella pneumoniae*: a major worldwide source and shuttle for antibiotic resistance. FEMS Microbiol Rev.

[122] Temkin E, Fallach N, Almagor J, Gladstone BP, Tacconelli E (2018). Estimating the number of infections caused by antibiotic-resistant *Escherichia coli* and *klebsiella pneumoniae* in 2014: a modelling study. Lancet Glob Health.

[123] Gu D, Dong N, Zheng Z, Lin D, Huang M (2018). A fatal outbreak of ST11 carbapenem-resistant hypervirulent *klebsiella pneumoniae* in a chinese hospital: a molecular epidemiological study. Lancet Infect Dis.

[124] Dong N, Zhang R, Liu L, Li R, Lin D (2018). Genome analysis of clinical multilocus sequence type 11 *klebsiella pneumoniae* from china. Microb Genom.

[125] Yao H, Qin S, Chen S, Shen J, Du XD (2018). Emergence of carbapenem-resistant hypervirulent *klebsiella pneumoniae*. Lancet Infect Dis.

[126] Wong MHY, Shum H-P, Chen JHK, Man M-Y, Wu A (2018). Emergence of carbapenem-resistant hypervirulent *Klebsiella pneumoniae*. Lancet Infect Dis.

[127] Xu M, Fu Y, Fang Y, Xu H, Kong H (2019). High prevalence of KPC-2-producing hypervirulent *Klebsiella pneumoniae* causing meningitis in eastern China. Infect Drug Resist.

