# Supplementary material for: Decentralized nanopore genomics reveals diverse Klebsiella pneumoniae and no evidence of patient–patient transmission in a New Zealand hospital
Source: Microb Genom. 2026 Apr 30;12(4):001700. doi: 10.1099/mgen.0.001700 (PMC13136631; doi:10.1099/mgen.0.001700)
Supplement: Uncited Fig. S1. [file mgen-12-01700-s001.pdf]

SUPPLEMENTARY APPENDIX

Decentralized nanopore genomics reveals diverse *Klebsiella pneumoniae* and no evidence of patient-patient transmission in a New Zealand hospital

1.1 Author names

Rhys T. White[1], Sarah Bakker[1], Megan Burton[2], Kristin Dyet[1], Juliet Elvy[1,3], Alexandra Eustace[1], Marissa P. Griffith[4,5], Samantha Hutton[2], Julianna Lees[1], Nathan J. Raabe[4,5,6], Brandon Su[2], Audrey Tiong[1], David J. Winter[1], Kelly L. Wyres[7,8,9], Max Bloomfield[2,10]

1.2 Affiliation(s)

[1]New Zealand Institute for Public Health and Forensic Science, Health Security, Porirua 5022, New Zealand

[2]Awanui Labs Wellington, Department of Microbiology and Molecular Pathology, Wellington 6021, New Zealand

[3]Awanui Labs Dunedin, Department of Microbiology and Molecular Pathology, Dunedin 9016, New Zealand

[4]University of Pittsburgh, Center for Genomic Epidemiology, Microbial Genomics Epidemiology Laboratory, Pittsburgh, PA 15261, USA

[5]University of Pittsburgh School of Medicine, Division of Infectious Diseases, Pittsburgh, PA 15261, USA

[6]University of Pittsburgh, School of Public Health, Department of Epidemiology, Pittsburgh, PA 15261, USA

[7]Monash University, Department of Infectious Diseases, School of Translational Medicine, Melbourne 3004, Australia

[8]Monash University, Centre to Impact AMR, Clayton 3800, Australia

[9]London School of Hygiene & Tropical Medicine, Department of Infection Biology, Faculty of Infectious and Tropical Diseases, London WC1E 7HT, United Kingdom

[10]Te Whatu Ora/Health New Zealand, Infection Services, Capital, Coast & Hutt Valley, Wellington 6021, New Zealand

1.3 Corresponding author and email address

*Corresponding author: Rhys White, New Zealand Institute for Public Health and Forensic Science, Porirua, New Zealand; Telephone: +64-4-914-0700; E-mail: rhys.white@phfscience.nz

This file includes the following:

Supplementary Figure S1. Overview of the nanopore-based genomic analysis workflow.

Supplementary Figure S2. Chromosomal tandem amplification of an IS*26*-Tn*3* composite module.

Supplementary Figure S3. Chromosome-wide single-nucleotide variant (SNV) distribution for *Klebsiella pneumoniae* sequence type (ST)253 isolates sharing an identical LIN code.

Supplementary Figure S4. Chromosome-wide single-nucleotide variant (SNV) distribution for *Klebsiella pneumoniae* sequence type (ST)557 isolates sharing an identical LIN code.

Supplementary Figure S5. Chromosome-wide single-nucleotide variant (SNV) distribution for *Klebsiella pneumoniae* sequence type (ST)3041 isolates sharing an identical LIN code

Supplementary Figure S6. Chromosome-wide single-nucleotide variant (SNV) distribution for *Klebsiella pneumoniae* sequence type (ST)133 isolates sharing an identical LIN code.

Supplementary Figure S7. Chromosome-wide single-nucleotide variant (SNV) distribution for *Klebsiella pneumoniae* sequence type (ST)90 isolates sharing an identical LIN code.

Supplementary Figure S8. Chromosome-wide single-nucleotide variant (SNV) distribution for *Klebsiella pneumoniae* sequence type (ST)252 isolates sharing an identical LIN code.

Supplementary Figure S9. Chromosome-wide single-nucleotide variant (SNV) distribution for *Klebsiella pneumoniae* sequence type (ST)950 isolates sharing an identical LIN code.

Supplementary Figure S10. Size distribution of plasmids by incompatibility group.

Supplementary Figure S11. Chromosomal versus plasmid-borne antimicrobial resistance determinants in 118 complete *Klebsiella pneumoniae* genomes.

Supplementary Figure S12. Chromosomal versus plasmid-borne virulence determinants in 118 complete *Klebsiella pneumoniae* genomes.

Supplementary Figure S13. Chromosomal integration of a plasmid-derived virulence region in *Klebsiella pneumoniae* kp221229_barcode02.

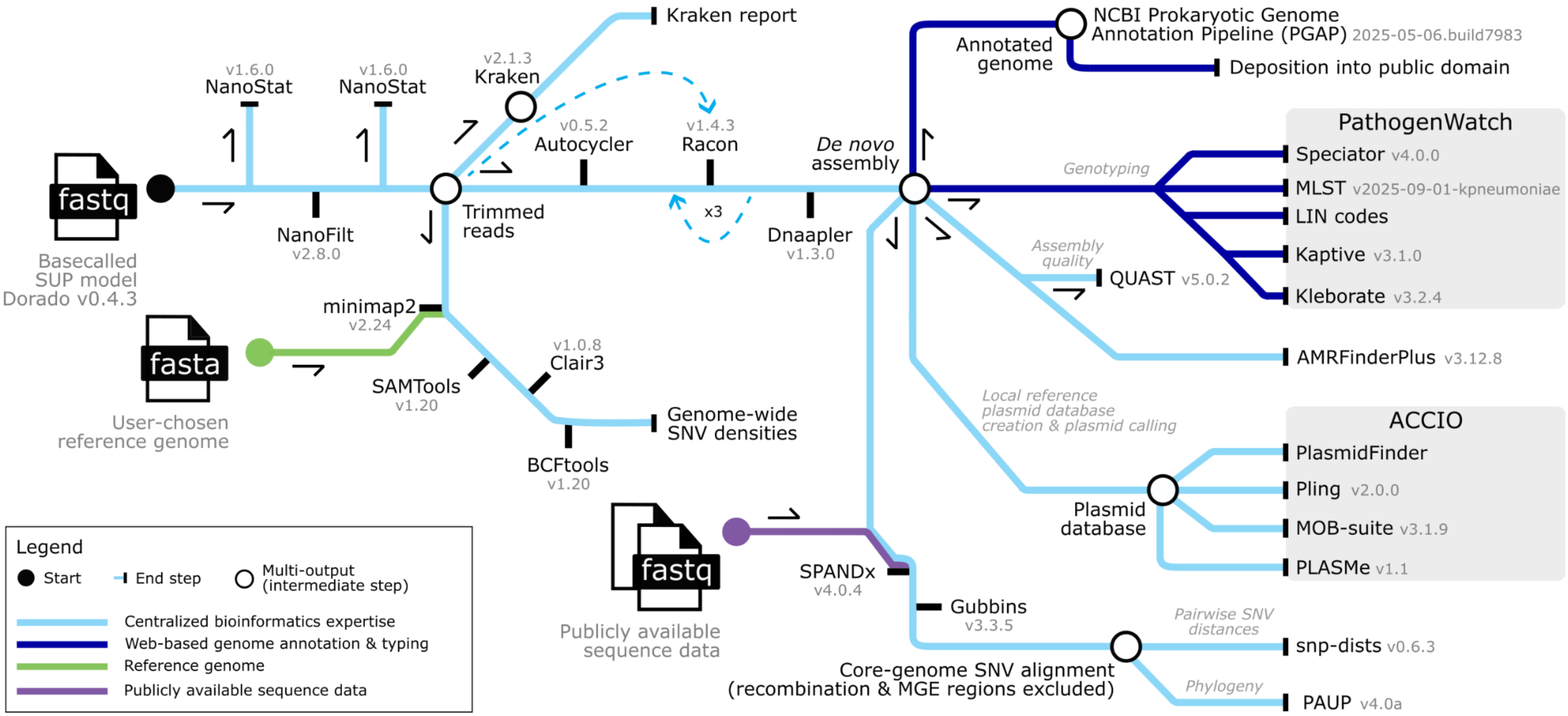

Supplementary Figure S1. Overview of the nanopore-based genomic analysis workflow. Schematic representation of the analytical workflow applied to *Klebsiella pneumoniae* isolates, from nanopore read processing through to genome reconstruction, genotyping, plasmid analysis, and comparative genomics. Basecalled nanopore reads were quality filtered and trimmed before analytical streams. Nanopore reads were assembled using Autocycler and polished with minimap2 and Racon, followed by reorientation with dnaapler to generate chromosomal and plasmid sequences. Assemblies were subsequently used for genome annotation (NCBI PGAP), genotyping and characterization (PathogenWatch), antimicrobial resistance detection (AMRFinderPlus), and plasmid analysis using the ACCIO framework. A reference-based approach was used to derive genome-wide single-nucleotide variant (SNV) densities via read mapping and variant calling. For global contextualization, publicly available paired-end sequence data (and simulated reads from nanopore assemblies; see SPANDx manual) were analyzed, with recombination filtered using Gubbins to generate a core-genome SNV alignment. Pairwise SNV distances and phylogenetic relationships were inferred using snp-dists and PAUP, respectively.

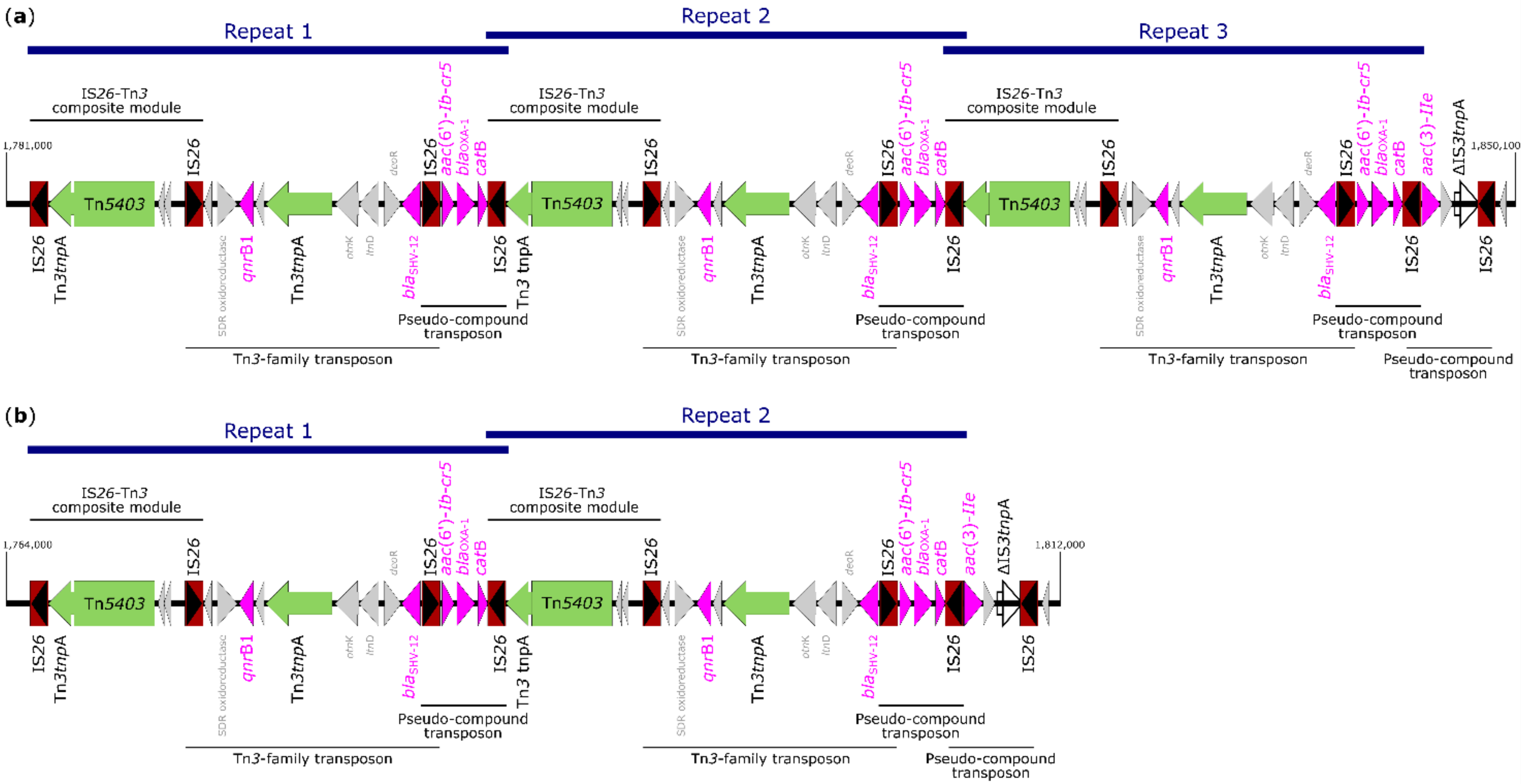

Supplementary Figure S2. Chromosomal tandem amplification of an IS*26*-Tn*3* composite module. (**a**) kp220713_barcode45 (GenBank: JBRENJ000000000): three repeats; (**b**) kp220713_barcode46 (GenBank: JBRENI000000000): two repeats. Each repeat contains *qnr*B1, $bla_{\mathrm{SHV\text{-}12}}$, *aac*(6′)-*Ib-cr5*, $bla_{\mathrm{OXA\text{-}1}}$, and *cat*B3; *aac*(3)-*IIe* is positioned just beyond the last repeat. IS*26* (red), Tn*3*-family transposase/Tn*5403* (green), genes conferring antimicrobial resistance (pink), and additional cargo (grey) are annotated. Genome coordinates are labelled.

.

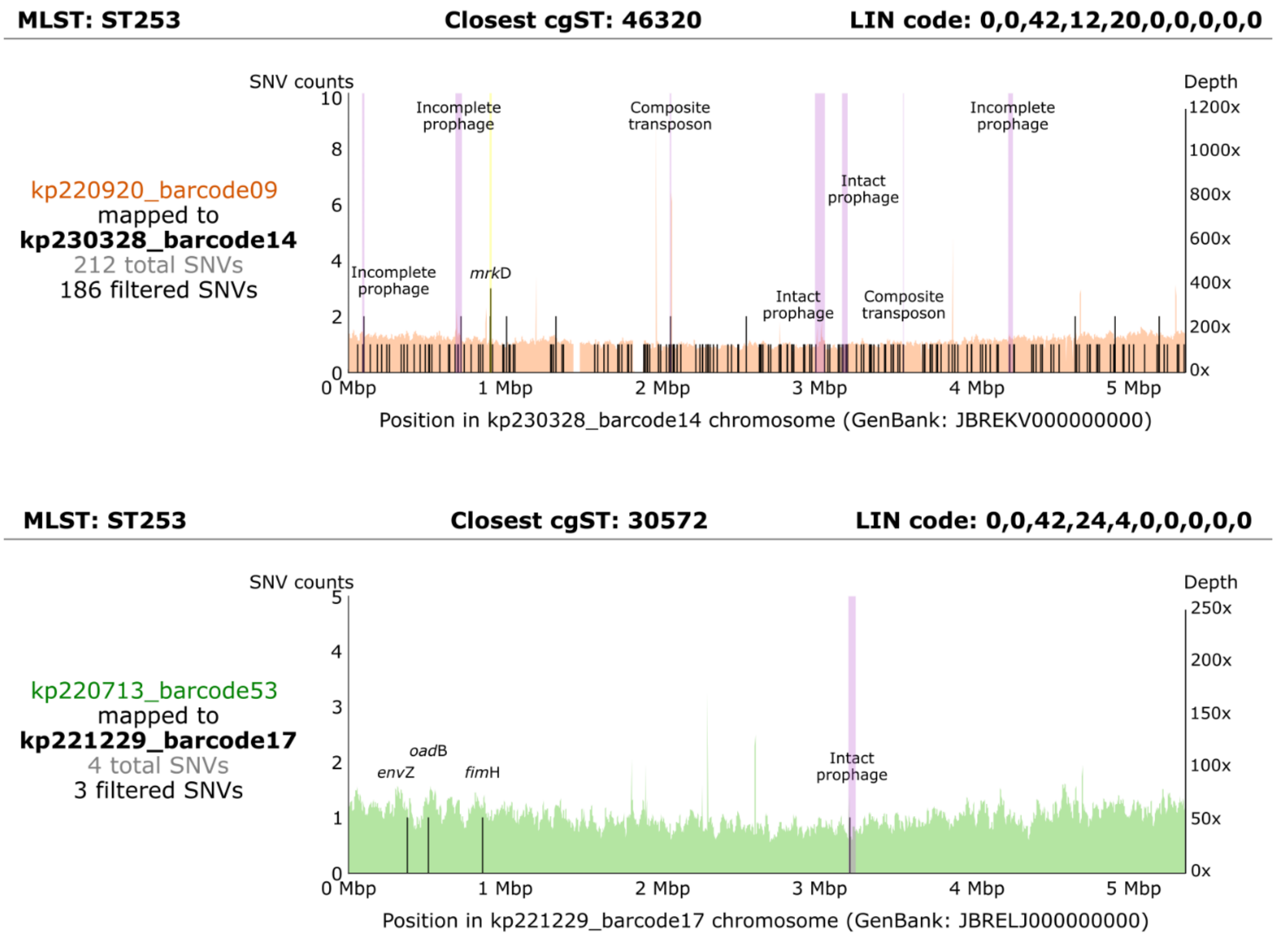

Supplementary Figure S3. **Chromosome-wide single-nucleotide variant (SNV) distribution for *Klebsiella pneumoniae* sequence type (ST)253 isolates sharing an identical LIN code**. (Top panel) Different patients, same ward, different months: kp220920_barcode09 (Medical Assessment and Planning Unit (MAPU), September 2022, Female 80+ years) vs reference kp230328_barcode14 (MAPU, March 2023, Female 80+ years). Bars show the number of SNVs per 1000 bp window across the kp230328_barcode14 reference chromosome; the overlaid trace shows the mean sequencing depth per 1000 bp window (orange; plotted on right axis). The median read depth was 144-fold (interquartile range: 128× to 161×; range 0× to 1162×). (Bottom panel) same patient, months apart: kp220713_barcode53 (7NW, July 2022, Male 18 to <30 years) vs reference kp221229_barcode17 (Emergency Department, December 2022). Bars show the number of SNVs per 1000 bp window across the kp221229_barcode17 reference chromosome; the overlaid trace shows the mean sequencing depth per 1000 bp window (green; plotted on right axis). The median read depth was 54-fold (interquartile range: 46× to 62×; range 28× to 215×). The purple shading represents mobile genetic elements. The yellow shading represents a flagged region of recombination. Multilocus sequence typing, MLST; core-genome sequence type, cgST; and Life Identification Number, LIN.

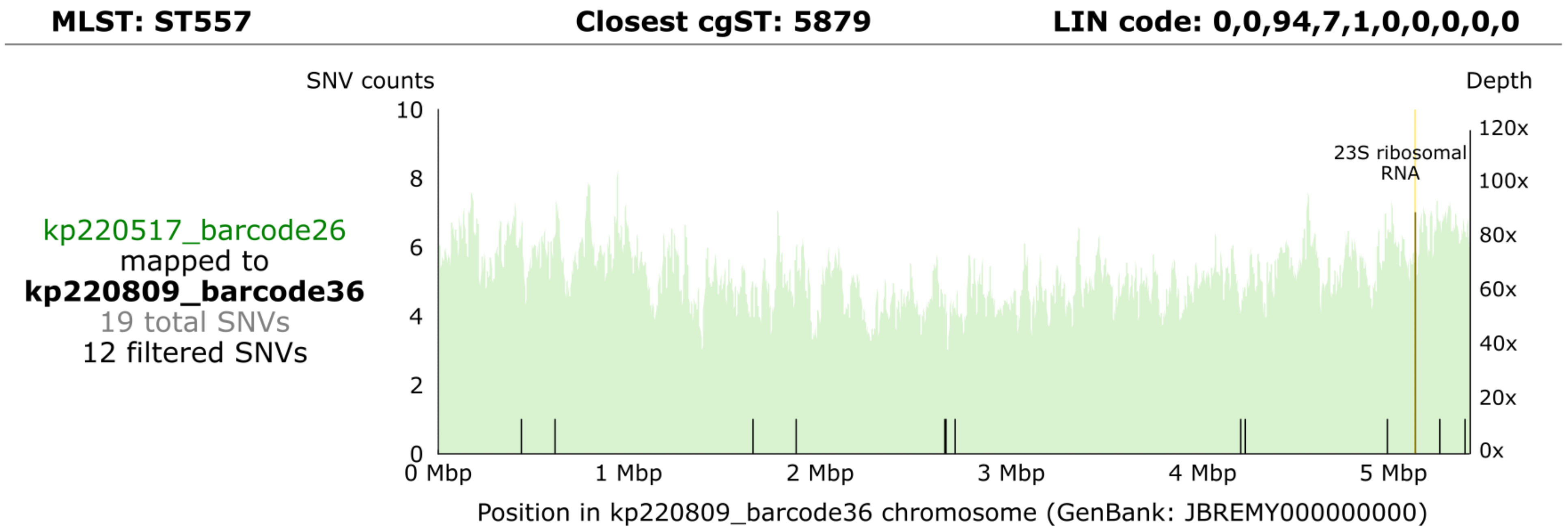

Supplementary Figure S4. **Chromosome-wide single-nucleotide variant (SNV) distribution for *Klebsiella pneumoniae* sequence type (ST)557 isolates sharing an identical LIN code**. Comparisons involve kp220517_barcode26 (Neonatal Intensive Care Unit, May 2022, Male 0 to <5 years) and kp220809_barcode36 (Surgical Assessment and Planning Unit, July 2022, Female 80+ years). Bars show the number of SNVs per 1000 bp window across the kp220809_barcode36 reference chromosome; the overlaid trace shows the mean sequencing depth per 1000 bp window (green; plotted on right axis). The median read depth was 66-fold (interquartile range: 59× to 75×; range 36× to 105×). The yellow shading represents a flagged region of recombination. Multilocus sequence typing, MLST; core-genome sequence type, cgST; and Life Identification Number, LIN.

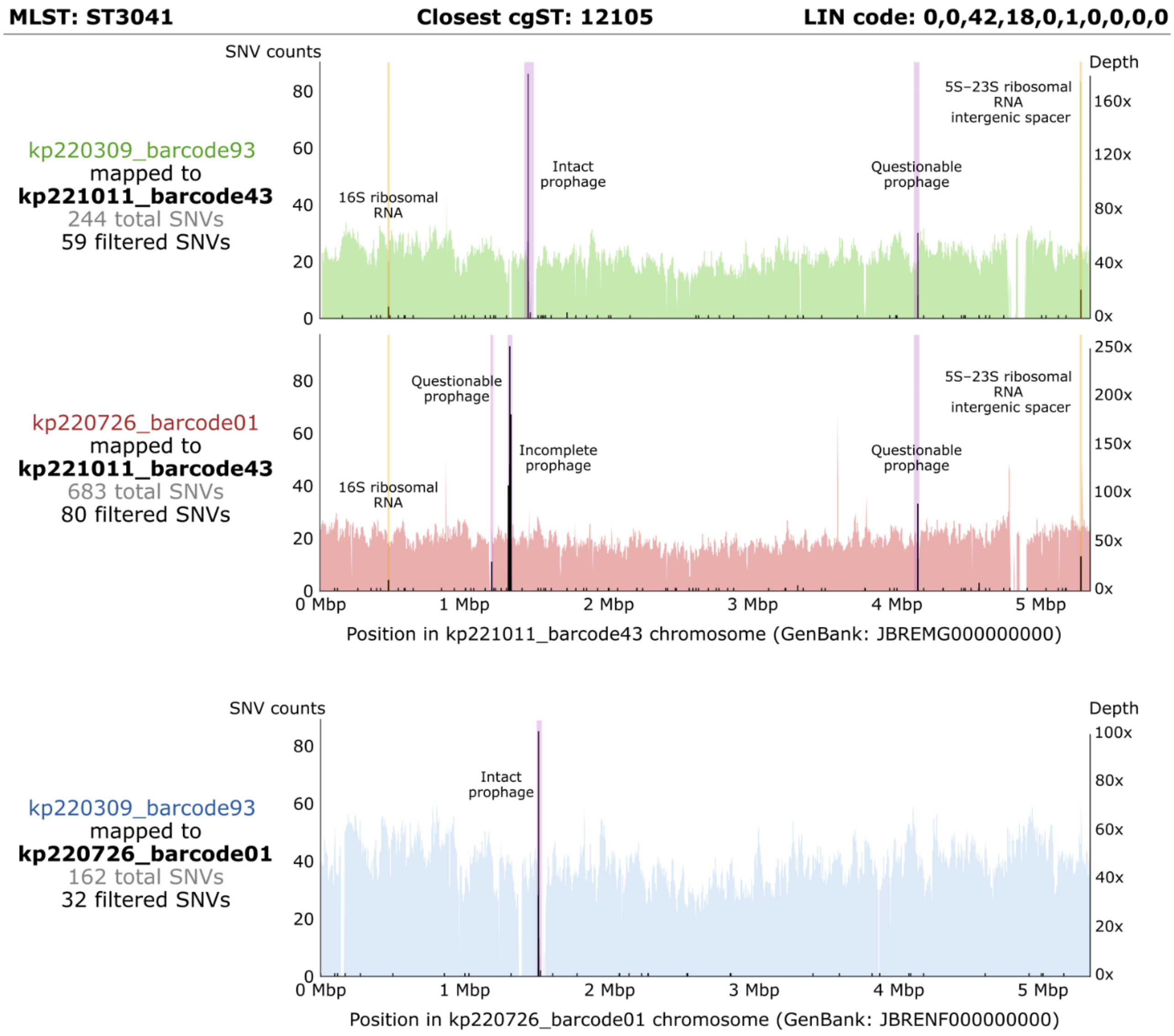

Supplementary Figure S5. **Chromosome-wide single-nucleotide variant (SNV) distribution for *Klebsiella pneumoniae* sequence type (ST)3041 isolates sharing an identical LIN code**. (Top) Bars show the number of SNVs per 1000 bp window across the kp221011_barcode43 reference chromosome; the overlaid trace shows the mean sequencing depth per 1000 bp window (green for kp220309_barcode93 and red for kp220726_barcode01; plotted on right axis). For kp220309_barcode93 (green), the median read depth was 47-fold (interquartile range: 40× to 53×; range 0× to 177×). For kp220726_barcode01 (red), the median read depth was 54-fold (interquartile range: 47× to 62×; range 0× to 203×). (Bottom) Bars show the number of SNVs per 1000 bp window across the kp220726_barcode01 reference chromosome; the overlaid trace shows the mean sequencing depth per 1000 bp window (blue; plotted on right axis). The median read depth was 47-fold (interquartile range: 41× to 53×; range 0× to 84×). The purple shading represents mobile genetic elements. The yellow shading represents a flagged region of recombination. Multilocus sequence typing, MLST; core-genome sequence type, cgST; and Life Identification Number, LIN.

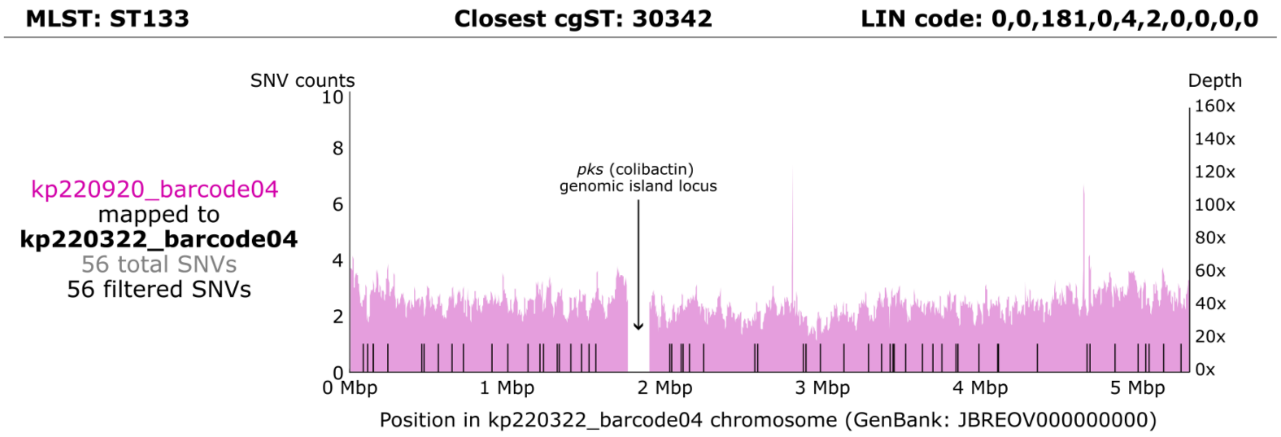

Supplementary Figure S6. **Chromosome-wide single-nucleotide variant (SNV) distribution for *Klebsiella pneumoniae* sequence type (ST)133 isolates sharing an identical LIN code**. Bars show the number of SNVs per 1000 bp window across the kp220322_barcode04 reference chromosome; the overlaid trace shows the mean sequencing depth per 1000 bp window (pink; plotted on right axis). The median read depth was 41-fold (interquartile range: 36× to 46×; range 0× to 153×). The *pks* (colibactin) genomic island locus is annotated at its position in the reference. Multilocus sequence typing, MLST; core-genome sequence type, cgST; and Life Identification Number, LIN.

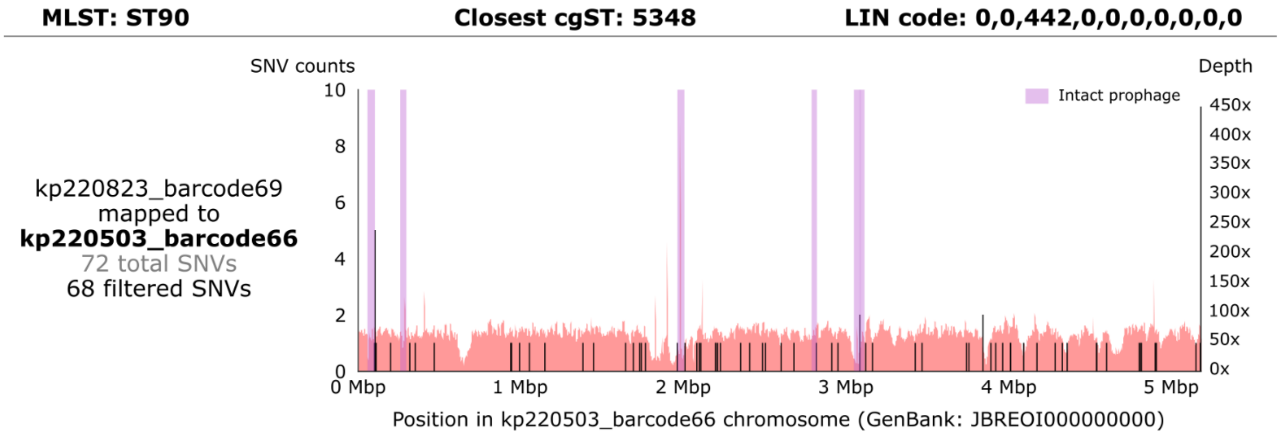

Supplementary Figure S7. **Chromosome-wide single-nucleotide variant (SNV) distribution for *Klebsiella pneumoniae* sequence type (ST)90 isolates sharing an identical LIN code**. Bars show the number of SNVs per 1000 bp window across the kp220503_barcode66 reference chromosome; the overlaid trace shows the mean sequencing depth per 1000 bp window (red; plotted on right axis). The median read depth was 65-fold (interquartile range: 57× to 72×; range 6× to 423×). The purple shading represents intact prophage elements. Multilocus sequence typing, MLST; core-genome sequence type, cgST; and Life Identification Number, LIN.

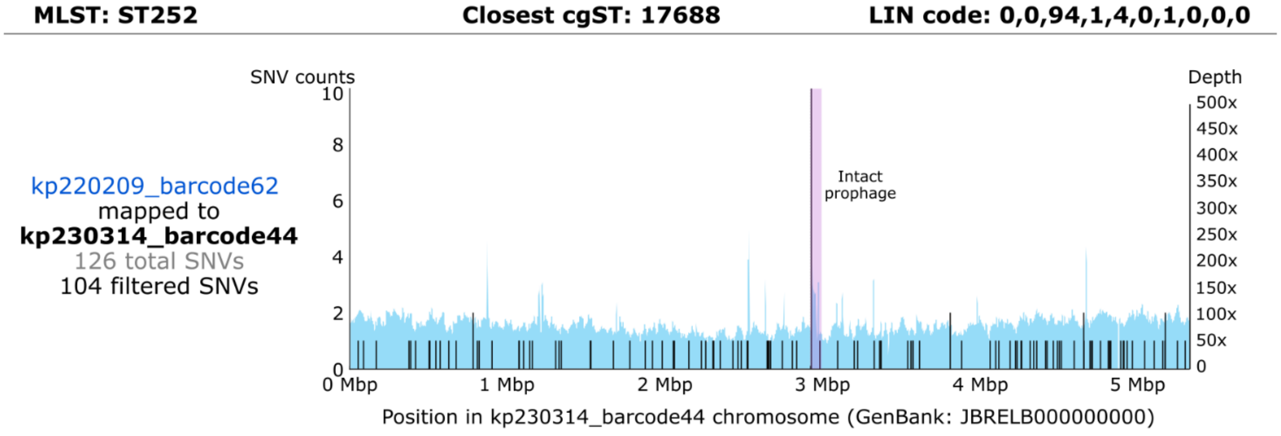

Supplementary Figure S8. **Chromosome-wide single-nucleotide variant (SNV) distribution for *Klebsiella pneumoniae* sequence type (ST)252 isolates sharing an identical LIN code**. Bars show the number of SNVs per 1000 bp window across the kp230314_barcode44 reference chromosome; the overlaid trace shows the mean sequencing depth per 1000 bp window (blue; plotted on right axis). The median read depth was 83-fold (interquartile range: 73× to 93×; range 0× to 499×). The purple shading represents an intact prophage element. Multilocus sequence typing, MLST; core-genome sequence type, cgST; and Life Identification Number, LIN.

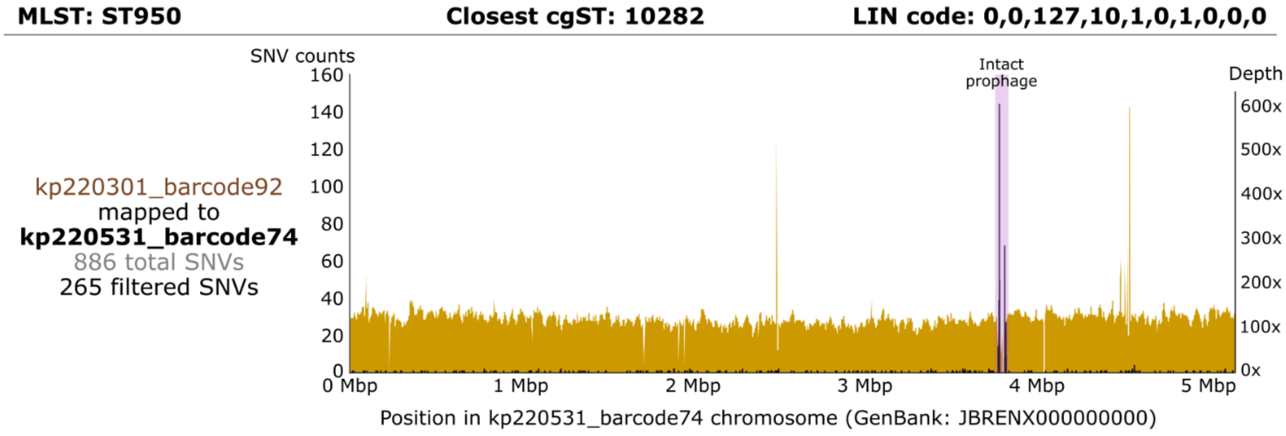

Supplementary Figure S9. **Chromosome-wide single-nucleotide variant (SNV) distribution for *Klebsiella pneumoniae* sequence type (ST)950 isolates sharing an identical LIN code**. Bars show the number of SNVs per 1000 bp window across the kp220531_barcode74 reference chromosome; the overlaid trace shows the mean sequencing depth per 1000 bp window (brown; plotted on right axis). The median read depth was 122-fold (interquartile range: 113× to 132×; range 0× to 1138×). The purple shading represents an intact prophage element. Multilocus sequence typing, MLST; core-genome sequence type, cgST; and Life Identification Number, LIN.

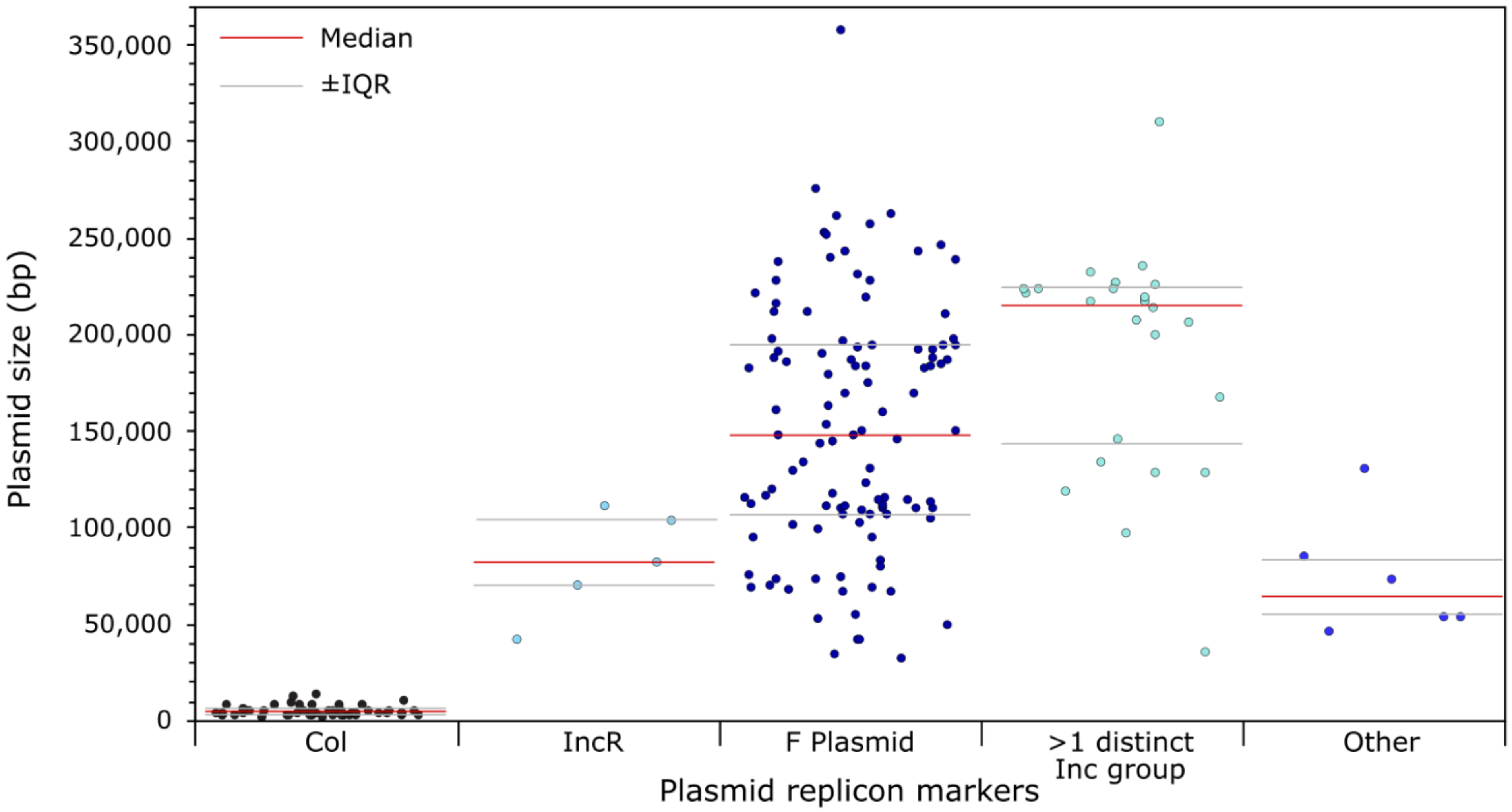

Supplementary Figure S10. Size distribution of plasmids by incompatibility group. Each dot represents an individual plasmid. Red lines indicate the median size, with grey bars showing the interquartile range (IQR). Col plasmids were consistently small, IncR plasmids showed intermediate sizes, F plasmids displayed the broadest range, and multi-replicon plasmids were typically large and mosaic.

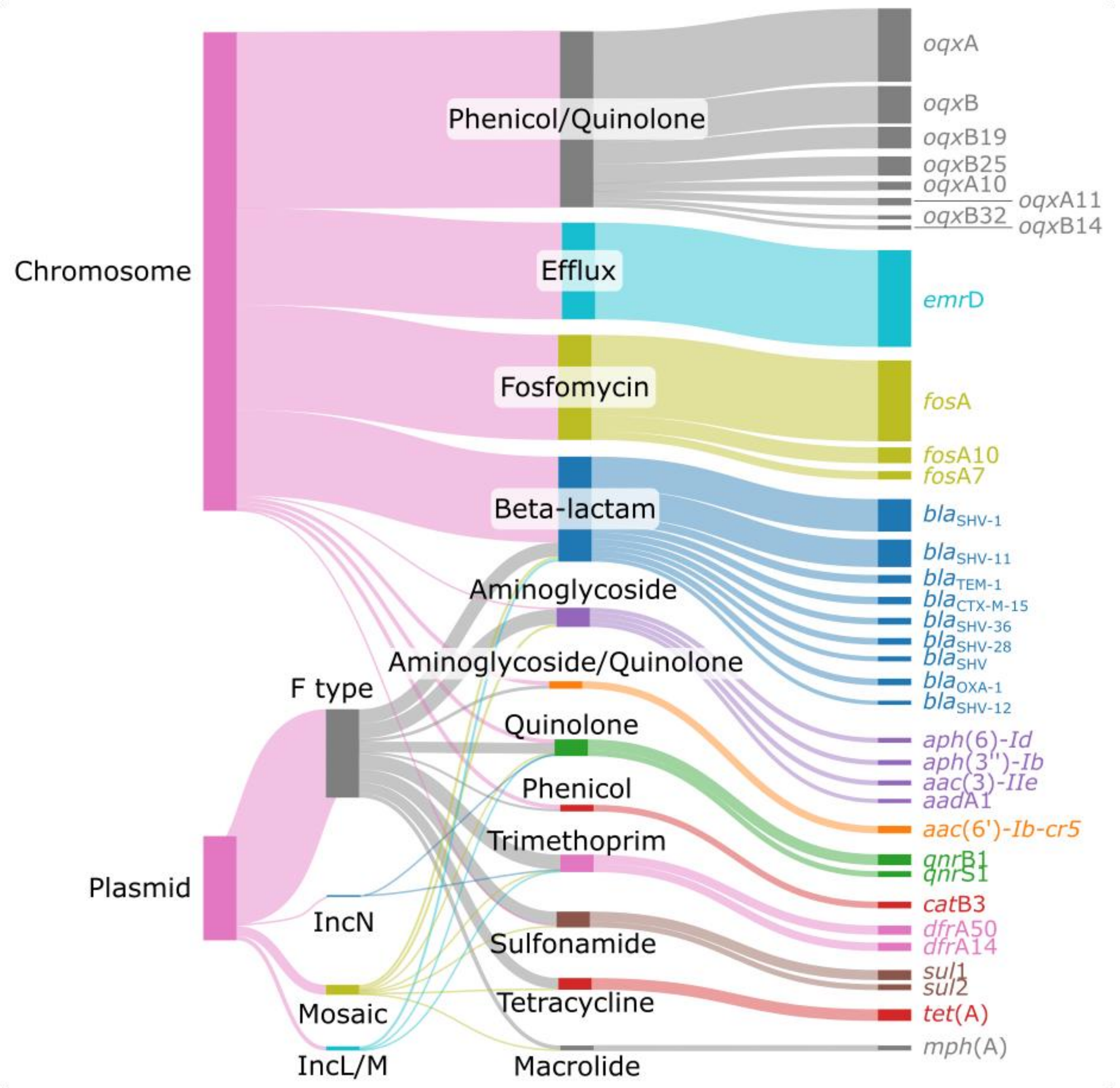

Supplementary Figure S11. Chromosomal versus plasmid-borne antimicrobial resistance determinants in 118 complete *Klebsiella pneumoniae* genomes. Sankey diagram shows antimicrobial resistance gene counts observed more than five times in the dataset, grouped by genomic location (left), functional class (middle), and individual gene (right). Multi-replicon plasmids are defined as mosaic. Chromosomal *bla*$_{SHV}$ represents intrinsic ampicillin resistance, whereas *fos*A, *oqx*AB, and *emr*D can modestly elevate minimum inhibitory concentrations but do not exceed clinical breakpoints (retained here for context but are not considered clinically relevant resistance determinants).

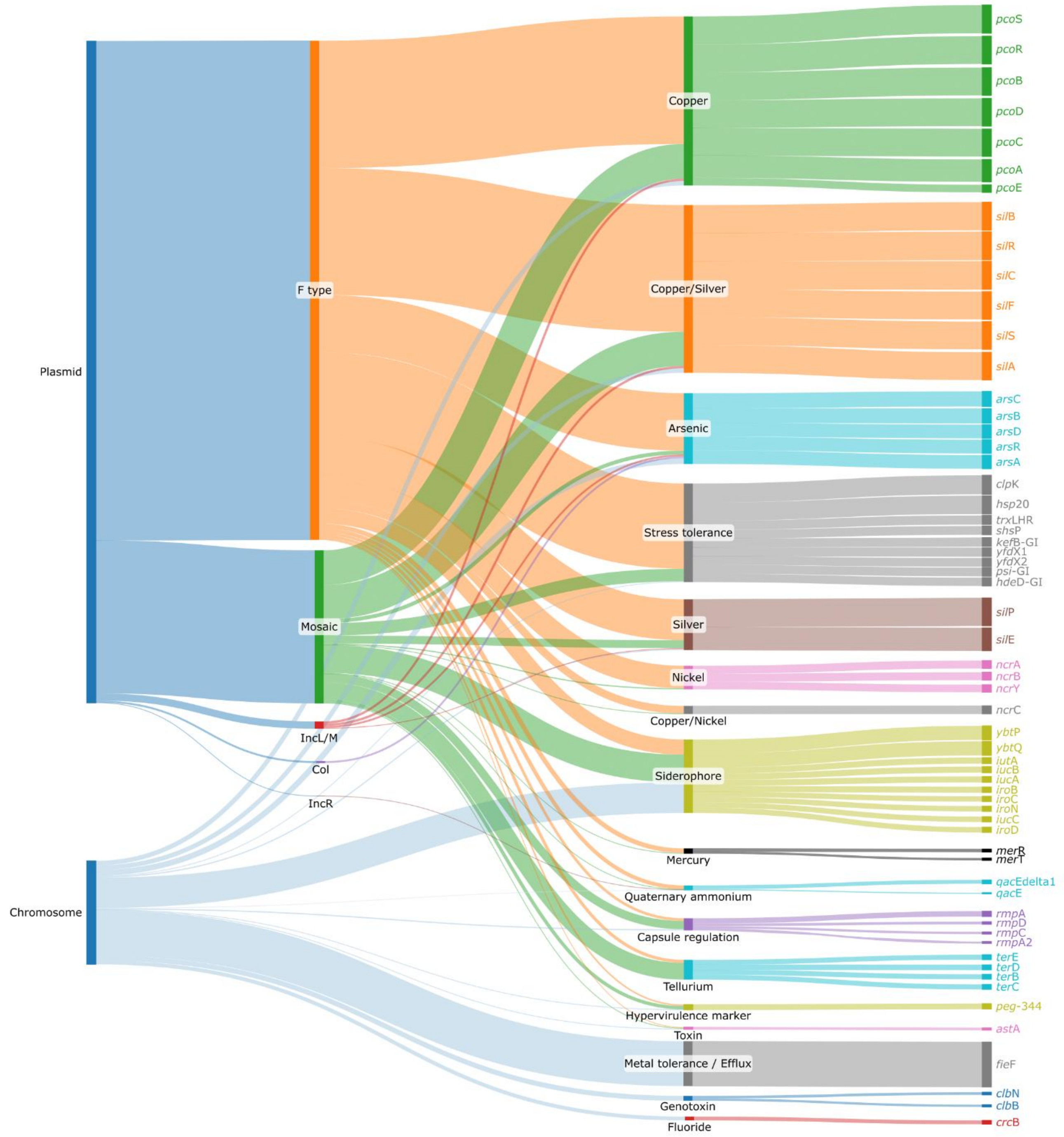

Supplementary Figure S12. Chromosomal versus plasmid-borne virulence determinants in 118 complete *Klebsiella pneumoniae* genomes. Sankey diagram shows virulence gene counts observed more than five times in the dataset, grouped by genomic location (left), functional class (middle), and individual gene (right). Multi-replicon plasmids are defined as mosaic. Most virulence determinants were plasmid-encoded, carried primarily on F-type and mosaic backbones.

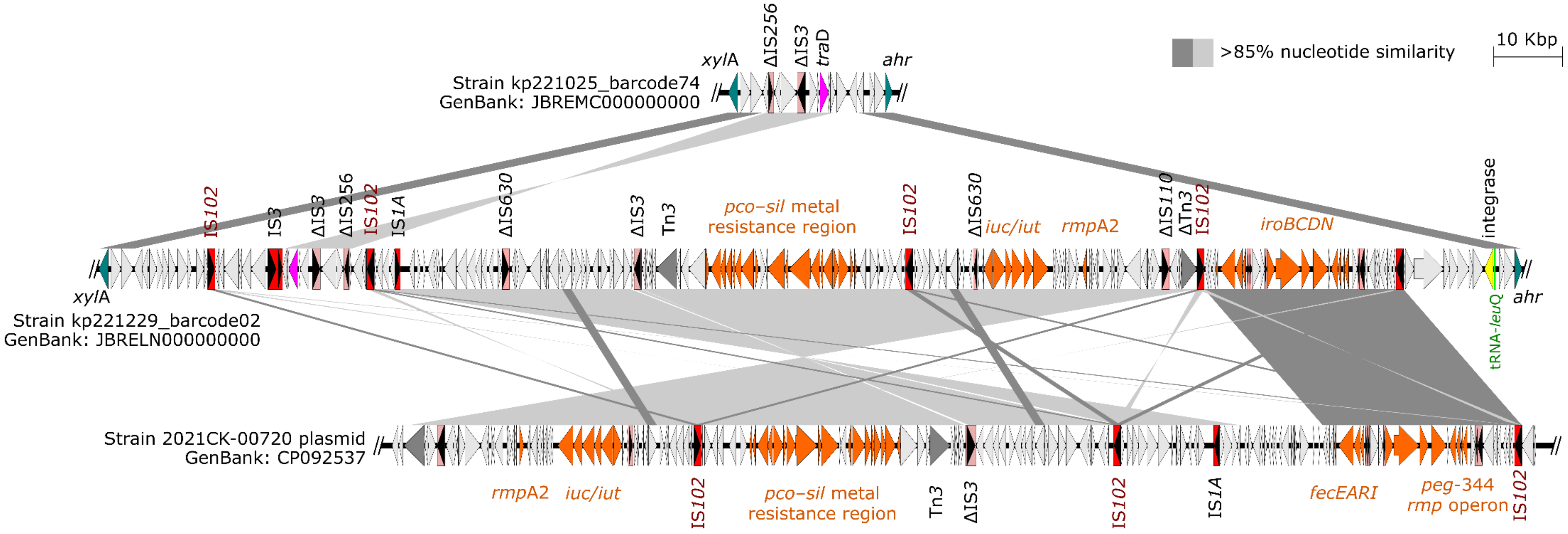

Supplementary Figure S13. Chromosomal integration of a plasmid-derived virulence region in *Klebsiella pneumoniae* kp221229_barcode02. Linear comparative alignment of the chromosomal region from *K. pneumoniae* kp221229_barcode02 with a reference virulence plasmid (CP092537). Grey shading indicates regions of >85% nucleotide similarity. The integrated chromosomal region in kp221229_barcode02 shows high synteny with the virulence plasmid backbone, including the aerobactin synthesis locus (*iuc*/*iut*), regulator *rmp*A2, and the salmochelin locus (*iro*BCDN). Multiple insertion sequences (including IS*102*, IS*3*, and IS*256*) flank and interrupt the integrated region. Gene orientation is indicated by arrows, and annotated mobile elements and virulence loci are labelled. Scale bar represents 10 kb.
